# Global prevalence and distribution of vancomycin resistant, vancomycin intermediate and heterogeneously vancomycin intermediate *Staphylococcus aureus* clinical isolates: a systematic review and meta-analysis

**DOI:** 10.1038/s41598-020-69058-z

**Published:** 2020-07-29

**Authors:** Aref Shariati, Masoud Dadashi, Majid Taati Moghadam, Alex van Belkum, Somayeh Yaslianifard, Davood Darban-Sarokhalil

**Affiliations:** 1 Department of Microbiology, School of Medicine, Iran University of Medical Sciences, Tehran, Iran; 2 Student Research Committee, Iran University of Medical Sciences, Tehran, Iran; 3 Department of Microbiology, School of Medicine, Alborz University of Medical Sciences, Karaj, Iran; 4 Non Communicable Diseases Research Center, Alborz University of Medical Sciences, Karaj, Iran; 5 Open Innovation and Partnerships, Route de Port Michaud, 38390 La Balme Les Grottes, France

**Keywords:** Microbiology, Health care

## Abstract

Vancomycin-resistant *Staphylococcus aureus* (VRSA), Vancomycin-intermediate *S. aureus* (VISA) and heterogeneous VISA (hVISA) are subject to vancomycin treatment failure. The aim of the present study was to determine their precise prevalence and investigate prevalence variability depending on different years and locations. Several international databases including Medline (PubMed), Embase and Web of Sciences were searched (data from 1997 to 2019) to identify studies that addressed the prevalence of VRSA, VISA and hVISA among human clinical isolates around the world. Subgroup analyses and meta-regression were conducted to indicate potential source of variation. Publication bias was assessed using Egger’s test. Statistical analyses were conducted using STATA software (version 14.0). Data analysis showed that VRSA, VISA and hVISA isolates were reported in 23, 50 and 82 studies, with an overall prevalence of 1.5% among 5855 *S. aureus* isolates, 1.7% among 22,277 strains and 4.6% among 47,721 strains, respectively. The overall prevalence of VRSA, VISA, and hVISA before 2010 was 1.2%, 1.2%, and 4%, respectively, while their prevalence after this year has reached 2.4%, 4.3%, and 5.3%. The results of this study showed that the frequency of VRSA, VISA and hVISA after 2010 represent a 2.0, 3.6 and 1.3-fold increase over prior years. In a subgroup analysis of different strain origins, the highest frequency of VRSA (3.6%) and hVISA (5.2%) was encountered in the USA while VISA (2.1%) was more prevalent in Asia. Meta-regression analysis showed significant increasing of VISA prevalence in recent years (*p* value ≤ 0.05). Based on the results of case reports (which were not included in the calculations mentioned above), the numbers of VRSA, VISA and hVISA isolates were 12, 24 and 14, respectively, among different continents. Since the prevalence of VRSA, VISA and hVISA has been increasing in recent years (especially in the Asian and American continents), rigorous monitoring of vancomycin treatment, it’s the therapeutic response and the definition of appropriate control guidelines depending on geographical regions is highly recommended and essential to prevent the further spread of vancomycin-resistant *S. aureus*.

## Introduction

*Staphylococcus aureus* is a common pathogen that causes various community and hospital-acquired diseases, including endocarditis, wound abscesses, osteomyelitis, skin and soft tissue infections, pneumonia and toxin-mediated syndromes in both healthy people and those with underlying illnesses^1,2^. Over the past 20 years, this bacterial species has developed resistance to many antibiotics, beta-lactams in particular^3,4^. During the seventies of the previous century, reports indicated that in the USA there was a significant increase in the morbidity and health care-associated costs, caused by methicillin-resistant *S. aureus* (MRSA)^4^. Currently, MRSA is endemic in hospitals around the world and the emergence of community-associated (CA) MRSA has added another serious concern^5^. Vancomycin, the first glycopeptide antibiotic to be discovered, provides one of the empiric therapies and still is a mainstay for treatment of MRSA infections^2^. In 1997, the first Vancomycin Intermediate *S. aureus* (VISA) with Minimum Inhibitory Concentration (MIC) of 8 μg/ml, was reported from Japan^6^. In 2002, the first case of Vancomycin-resistant *S. aureus* (VRSA) was reported in a diabetic patient in the USA^7^. Previously, in vitro studies suggested the existence of various mechanisms for vancomycin resistance in MRSA, the main one being the decreased permeability and the increased thickness of the cell wall and hence a decreased availability of vancomycin for intracellular target molecules. Another type of resistance was caused by plasmid-mediated vancomycin resistance genes (*vanA, vanB, vanD, vanE, vanF,* and *vanG*) which may have been transferred from enterococcal species^6,8–10^. Besides, a recent study has shown that VISA growth rate is lower and that the cells harbor a thicker cell wall than those fully susceptible.^9,11^. Heterogeneous VISA (hVISA) show MICs in the susceptible range (≤ 2 μg/mL), but they contain a sub-population that expresses a resistant phenotype^12,13^. Infections caused by VISA and hVISA lead to higher rates of vancomycin treatment failure and are associated with extended hospitalization, higher risk of persistent infection, and elevated treatment costs^13,14^. Despite a published systematic review and meta-analysis study on the prevalence VISA and hVISA^15^ 5 years ago, there has not been published a comprehensive study on the prevalence VRSA, VISA and hVISA worldwide, yet. In the present systematic review and meta-analysis, we pooled published studies that reported the prevalence of VRSA, VISA and hVISA. The findings of the current study will more precisely define the current epidemiology of VRSA, VISA and hVISA and may help to develop more appropriate antibiotic stewardship policies to combat vancomycin resistance.

## Methods

### Search strategy

A comprehensive systematic literature search was performed in Medline (via PubMed), Embase, and Web of Science databases for original research articles published from 1997 until September 2019. The following terms were applied in our search strategy: *Staphylococcus aureus, S. aureus*, Vancomycin Resistant *Staphylococcus aureus*, Vancomycin Resistant *S. aureus*, VRSA, Vancomycin Intermediate *Staphylococcus aureus*, Vancomycin Intermediate *S. aureus*, VISA, heterogeneous Vancomycin Intermediate *Staphylococcus aureus*, heterogeneous Vancomycin Intermediate *S. aureus* and hVISA. We also searched the bibliographies of relevant articles to identify additional studies.

### Inclusion and exclusion criteria

All original human studies on the prevalence of VRSA, VISA and hVISA among clinical *S. aureus* isolates that reported sufficient data (including prevalence, evaluation methods, and country of origin) were assessed^5,12,13,16–197^. Titles, abstracts and full texts of the recorded studies were checked based on the inclusion and exclusion criteria. The exclusion criteria were: (1) animal research only, (2) studies considering vancomycin-resistant bacteria beyond *S. aureus,* (3) reviews, (4) abstracts presented in conferences, and (5) duplicate studies. Two of the authors (AS and MT) evaluated all studies based on inclusion and exclusion criteria and selected the appropriate papers.

### Data extraction and definitions

The following items were extracted from each included study: the last name of the first author, study years, time of publication, country, number of VRSA, VISA and hVISA, number of patients with staphylococcal infection, phenotypic methods used, genotypic identification methods applied and the sample source. Data were collected by two independent examiners and verified by another researcher. According to the CLSI, the definition of VRSA, VISA and hVISA in *S. aureus* isolates with reduced susceptibility to vancomycin is MIC ≥ 16 μg/mL, MIC of 4–8 μg/mL; and MIC of 1–2 μg/mL, respectively. Furthermore, articles before 2006 used the old definition of VRSA and VISA (VRSA, MIC ≥ 32 μg/mL; VISA, MIC of 8–16 μg/mL)^198^.

### Quality assessment

All reviewed articles were evaluated for quality (according to guidelines developed by the Joanna Briggs Institute), and only high-quality articles that met those rules were included^199^.

### Meta-analysis

Statistical analysis was conducted with STATA software, version 14.0 (Stata Corporation, College Station, Texas, USA) to report the global prevalence of VRSA, VISA and hVISA isolated from human clinical samples. The data were pooled using the fixed-effects (FEM)^200^ and the random-effects model (REM). Subgroup analyses were conducted based on the type of isolates, publication year and geographic areas (continent/countries). Statistical heterogeneity was evaluated using the Q-test and the I2 statistical methods^201^. *P* value < 0.1 was regarded as statistically-significant^202^. To assess possible publication bias, we calculated the Egger’s test.

### Meta-regression analysis

We evaluated whether the prevalence of VRSA, VISA and hVISA changed over time by performing restricted maximum likelihood (REML) random effect meta-regression analysis based on publication year as the moderator. A *p* value less than 0.05 (*p* value ≤ 0.05) was considered statistically significant.

## Results

### Characteristics of included studies

**Figure 1:**
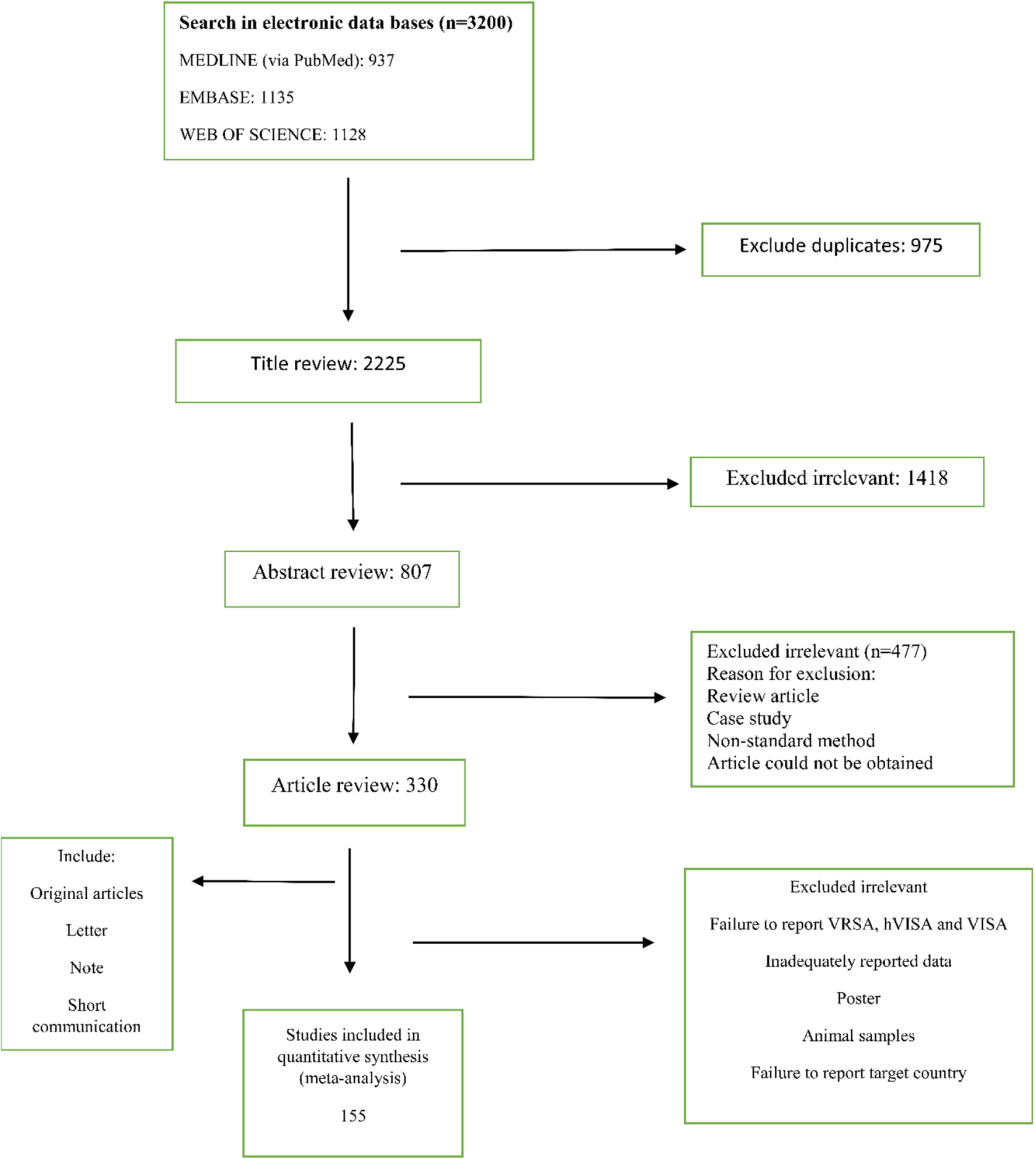
Flow chart of publication selection and their inclusion in the systematic review.

In total, 3200 citations were recorded in the initial database searches. Since we collected data from three databases, many duplicate studies were included. After removing 975 duplicates, titles and abstracts of 2225 articles were checked and 1418 irrelevant studies not meeting the Briggs Institute rules were excluded from our review. In the next screening, 477 non-relevant studies were removed upon reading the full text. In the end, 155 articles were included in the final analysis (Fig. 1 shows a flow chart).

### The prevalence of VRSA, VISA and hVISA isolates among human clinical isolates

Out of 155 articles that reported the prevalence of VRSA, VISA and hVISA, 89 studies were from Asia, 22 from Europe, 31 from America, 9 from Africa and 4 from Oceania (Table 1). Data analysis showed that VRSA, VISA and hVISA isolates were reported in 23, 50 and 82 studies, with an overall prevalence of 1.5% [(95% CI) 1.0–2.0] among 5855 *S. aureus* isolates, 1.7% [(95% CI) 1.3–2.0] among 22,277 strains and 4.6% [(95% CI) 4.1–5.1] among 47,721 strains, respectively (Table 2).

**Table 1:** Prevalence of VRSA, VISA and hVISA isolated from human clinical samples in different continents/countries based on published original studies.

Subgroup	Isolates	Country	Prevalence% (95% CI)	Number of studies	*P* value	I-squared	Tau-squared
Asia	VRSA	Overall	1.3 (0.8–1.9)	18	0.000	63.7	*P* < 0.001
		Iran	1.3 (0.5–2.0)	10	0.007	60.2	*P* < 0.001
		India	1.6 (0.3–2.8)	5	0.008	70.9	*P* < 0.001
		Bangladesh	4.5 (0.0–10.7)	1	0.727	0.0	*P* < 0.001
		Pakistan	3.3 (0.5–6.2)	1	0.727	0.0	*P* < 0.001
		Jordan	4.0 (0.6–7.4)	1	0.023	0.0	*P* < 0.001
	VISA	Overall	2.1 (1.6–2.6)	28	0.000	88.3%	*P* < 0.001
		China	0.5 (0.1–1.0)	2	0.960	0.0	*P* < 0.001
		India	4.6 (1.9–7.2)	6	0.000	88.3	*P* < 0.001
		Iran	3.6 (1.4–5.7)	6	0.000	77.7	*P* < 0.001
		Japan	0.6 (0.0–1.3)	3	0.074	61.6	*P* < 0.001
		Korea	0.7 (0.1–1.3)	2	0.626	0.0	*P* < 0.001
		Pakistan	5.6 (1.9–9.2)	3	0.000	87.6	*P* < 0.001
		Saudi-Arabia	18.0 (11.9–24.1)	1	–	–	*P* < 0.001
		Singapore	12.5 (3.8–21.2)	1	–	–	*P* < 0.001
		Taiwan	1.9 (0.0–4.0)	3	0.000	95.7	*P* < 0.001
		Thailand	0.3 (0.0–0.7)	1	–	–	*P* < 0.001
	hVISA	Overall	4.7 (3.9–5.4)	43	0.000	92.6%	*P* < 0.001
		China	10.0 (5.5.14.4)	6	0.000	90.9	*P* < 0.001
		Singapore	3.0 (0.2–5.8)	2	0.370	0.0	*P* < 0.001
		Taiwan	1.5 (0.4–2.6)	5	0.001	78.9	*P* < 0.001
		Thailand	9.7 (9.6–15.7)	5	0.000	96.2	*P* < 0.001
		Japan	8.4 (5.3–11.4)	5	0.001	79.9	*P* < 0.001
		Korea	3.3 (2.1–4.5)	8	0.000	91.4	*P* < 0.001
		Lebanon	4.4 (0.6–8.2)	1	–	–	*P* < 0.001
		India	2.5 (0.5–5.0)	4	0.006	76.2	*P* < 0.001
		Malaysia	2.5 (1.0–4.0)	3	0.594	0.0	*P* < 0.001
		Philippines	3.6 (0.0–10.4)	1	–	–	*P* < 0.001
		Vietnam	4.4 (0.0–8.8)	2	0.235	29.1	*P* < 0.001
		Israel	6.1 (3.2–8.9)	1	–	–	*P* < 0.001
Europe	VRSA	Overall	1.1 (0.0–2.7)	1	–	–	*P* < 0.001
		Italy	1.1 (0.0–2.7)	1	–	–	*P* < 0.001
	VISA	Overall	1.8 (0.8–2.8)	6	0.027	60.5%	*P* < 0.001
		Belgium	2.5 (1.1–4.0)	1	–	–	*P* < 0.001
		France	2.2 (0.5–3.8)	2	0.87	65.8	*P* < 0.001
		Turkey	2.7 (0.0–6.5)	1	–	–	*P* < 0.001
		Germany	0.7 (0.0–1.6)	1	–	–	*P* < 0.001
		Italy	1.4 (0.0–3.2)	1	–	–	*P* < 0.001
	hVISA	Overall	4.4 (3.2–5.5)	15	0.000	97.4%	*P* < 0.001
		France	5.9 (0.0–16)	2	0.000	99.5	*P* < 0.001
		Belgium	0.2 (0.1–0.4)	3	0.479	0.0	*P* < 0.001
		Ireland	2.5 (1.9–3.0)	1	–	–	*P* < 0.001
		Italy	6.9 (2.5–11.3)	4	0.000	85.4	*P* < 0.001
		Poland	4.9 (0.7–9.0)	1	–	–	*P* < 0.001
		Turkey	11.4 (1.7–21.1)	3	0.000	93.4	*P* < 0.001
		UK	3.4 (2.7–4.1)	1	–	–	P < 0.001
America	VRSA	Overall	3.6 (0.5–6.6)	1	–	–	*P* < 0.001
		Brazil	3.6 (0.5–6.6)	1	–	–	*P* < 0.001
	VISA	Overall	1.0 (0.5–1.4)	9	0.023	55.0%	*P* < 0.001
		USA	0.9 (0.5–1.3)	7	0.054	51.5	*P* < 0.001
		Brazil	4.1 (1.0–7.3)	2	0.352	0.0	*P* < 0.001
	hVISA	Overall	5.2 (4.3–6.1)	21	0.000	96.1%	*P* < 0.001
		USA	4.7 (3.7–5.6)	16	0.000	96.6	*P* < 0.001
		Brazil	17.3 (3.9–30.7)	3	0.000	88.4	*P* < 0.001
		Canada	5.3 (3.3–7.3)	1	–	–	*P* < 0.001
		Argentina	3.3 (0.0–6.9)	1	–	–	*P* < 0.001
Africa	VRSA	Overall	2.5 (0.1–4.8)	3	0.061	64.2	*P* < 0.001
		Algeria	1.4 (0.0–2.9)	1	–	–	*P* < 0.001
		Egypt	5.5 (2.3–8.7)	1	–	–	*P* < 0.001
		Nigeria	1.4 (0.0–4.0)	1	–	–	*P* < 0.001
	VISA	Overall	1.8 (0.1–3.4)	5	0.003	75.0%	*P* < 0.001
		Algeria	0.6 (0.1–1.1)	3	0.761	0.0	*P* < 0.001
		Kenya	4.2 (0.6–7.9)	1	–	–	*P* < 0.001
		Nigeria	15.1 (6.9–23.3)	1	–	–	*P* < 0.001
	hVISA	Overall	4.0 (0.2–7.8)	1	–	–	*P* < 0.001
		Egypt	4.0 (0.2–7.8)	1	–	–	*P* < 0.001
Oceania	VRSA	NR	NR	–	–	–	*P* < 0.001
	VISA	Overall	0.7 (0.0–1.3)	2	0.332	0.0%	*P* < 0.001
		Australia	0.7 (0.0–1.3)	2	0.332	0.0	*P* < 0.001
	hVISA	Overall	11.2 (8.3–14.1)	2	0.637	0.0	*P* < 0.001
		Australia	11.2 (8.3–14.1)	2	0.637	0.0	*P* < 0.001

Tau-squared: the extent of variation among the effects observed in different studies; I-squared: the percentage of variance in a meta-analysis that shows study heterogeneity.

**Table 2:** Prevalence of VRSA, VISA and hVISA isolated from human clinical samples based on two study periods.

Subgroup	Isolates	Prevalence% (95% CI)	Number of studies	*P* value	I-squared (%)	Tau-squared
Overall	VRSA	1.5 (1.0–2.0)	23	0.000	65.3	0.0001
	VISA	1.7 (1.3–2.0)	50	0.000	83.	0.0001
	hVISA	4.6 (4.1–5.1)	82	0.000	89.2	0.0003
Research before 2010	VRSA	1.2 (0.5–1.8)	8	0.017	59.0	0.0000
	VISA	1.2 (0.9–1.5)	31	0.000	79.0	0.0000
	hVISA	4.0 (2.9–5.0)	70	0.000	84.8	0.0003
Research after 2010	VRSA	2.4 (1.4–3.5)	15	0.000	69.0	0.0002
	VISA	4.3 (3.0–5.7)	19	0.000	86.3	0.0005
	hVISA	5.3 (2.5–8.3)	12	0.000	84.3	0.0003

### The prevalence of VRSA, VISA and hVISA in two study periods

**Figure 2:**
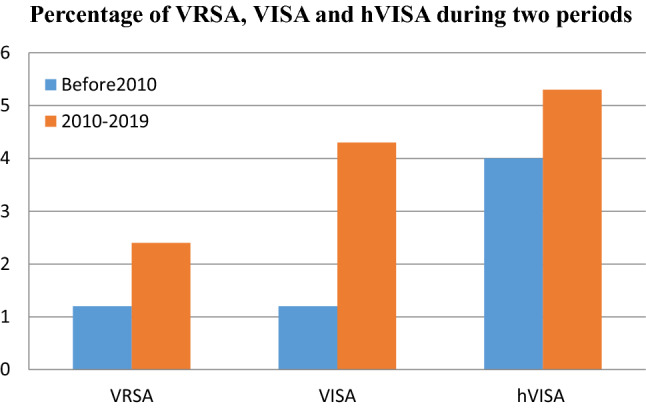
Distribution of VRSA, VISA and hVISA isolates among different continents based on published original and case report studies.

To analyze the trends for changes in the prevalence of VRSA, VISA and hVISA in more recent years, we performed a subgroup analysis for two periods (before 2010 and 2010–2019) (Table 2, Fig. 2). The prevalence of VRSA, VISA and hVISA gradually increased. Before 2010, the prevalence was 1.2% (95% CI 0.5–1.8) among 2444 *S. aureus* isolates, 1.2% (95% CI 0.9–1.5) among 18,469 isolates and 4.0 (95% CI 2.9–5.0) among 41,190 *S. aureus*, respectively. Prevalence reached 2.4% (95% CI 1.4–3.5) among 3411 *S. aureus* isolates, 4.3% (95% CI 3.0–5.7) among 3808 isolates and 5.3% (95% CI 1.8–4.1) among 6531 isolates in 2010–2019, respectively. The changes in VRSA, VISA and hVISA prevalence between periods are presented in Table 2. The results of this review indicate that the frequency of VRSA, VISA and hVISA after 2010 represent a 2.0, 3.6 and 1.3-fold increase over the prior years (Fig. 2).

**Figure 3:**
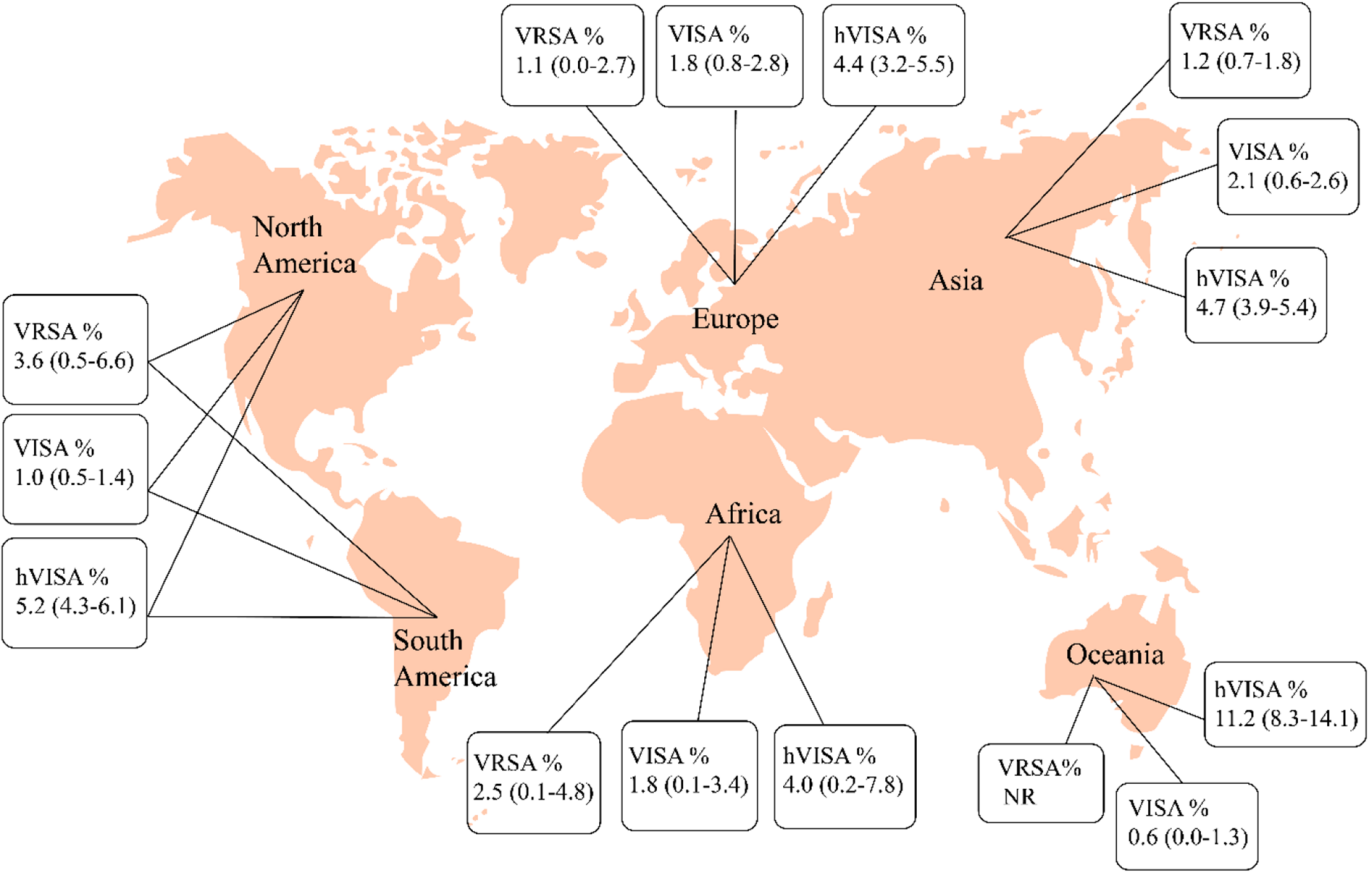
Distribution of VRSA, VISA and hVISA isolates among different countries based on meta-analysis of published original articles.

**Figure 4:**
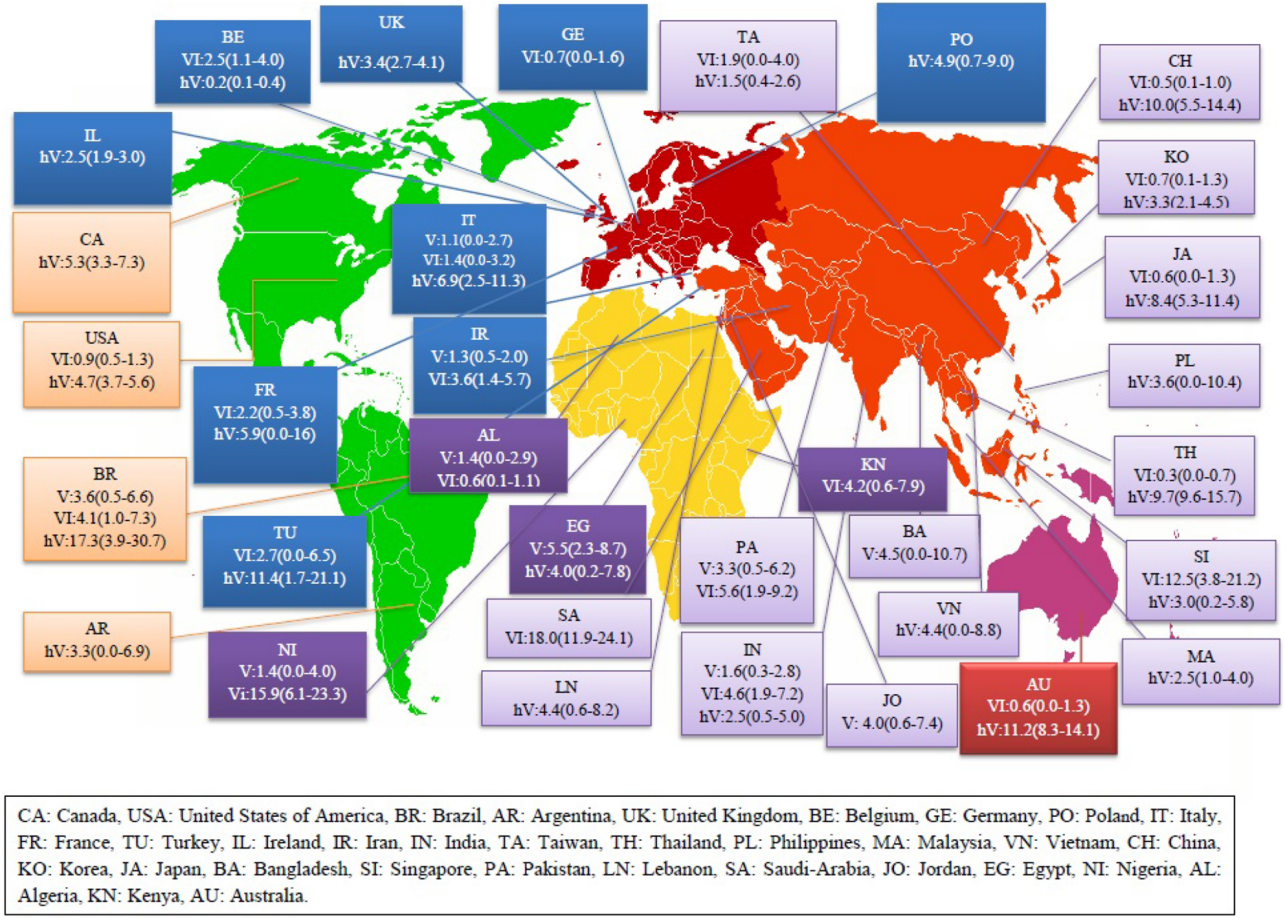
Distribution of VRSA, VISA and hVISA isolates among different countries based on meta-analysis of published original articles.

**Figure 5:**
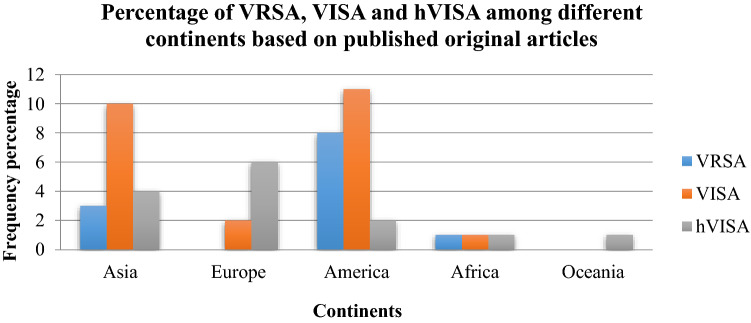
The number of VRSA, VISA and hVISA isolates among different countries based on meta-analysis of published original articles.

**Figure 6:**
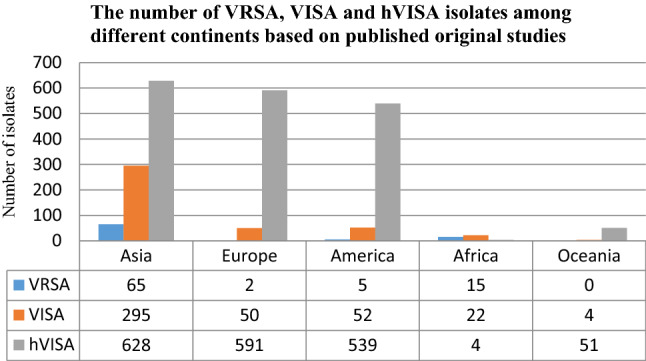
Prevalence of VRSA, VISA and hVISA isolated from human clinical samples based on two study periods.

### The prevalence of VRSA, VISA and hVISA on different continents

The prevalence of VRSA was 1.2% (95% CI 0.7–1.8) among 5043 *S. aureus* isolates in Asia, 1.1% (95% CI 0.0–2.7) among 179 isolates in Europe, 3.6% (95% CI 0.5–6.6) among 140 isolates in America and 2.5% (95% CI 0.1–4.8) among 493 isolates in Africa. There has been no report of VRSA from Oceania (Figs. 3, 4, 5, 6). The results of this review showed that the prevalence of VISA isolates was 2.1% (95% CI 1.6–2.6) among 13,449 *S. aureus* isolates, 1.8% (95% CI 0.8–2.8) among 2198 isolates, 1.0% (95% CI 0.5–1.4) among 5040 isolates, 1.8% (95% CI 0.1–3.4) among 1072 isolates and 0.6% (95% CI 0.0–1.3) among 518 isolates from Asia, Europe, America, Africa and Oceania, respectively (Figs. 3, 4, 5, 6). Moreover, the prevalence of hVISA in Asia, Europe, America, Africa and Oceania were 4.7% (95% CI 3.9–5.4) among 16,955 *S. aureus* isolates, 4.4% (95% CI 3.2–5.5) among 14,680 isolates, 5.2% (95% CI 4.3–6.1) among 15,532 isolates, 4.0% (95% CI 0.2–7.8) among 100 isolates and 11.2% (95% CI 8.3–14.1) among 454 isolates, respectively (Figs. 3, 4, 5, 6).

### The prevalence of VRSA, VISA and hVISA on different continents based on case reporting

**Figure 7:**
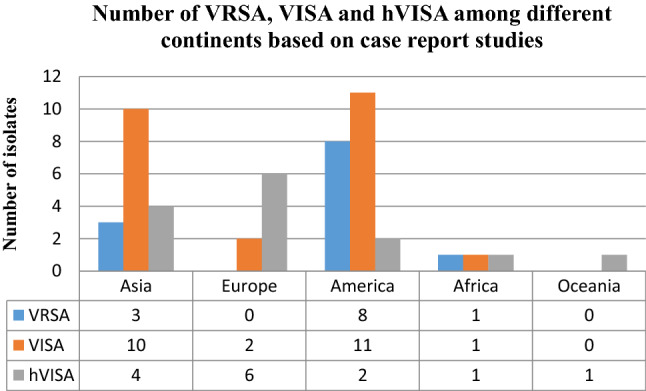
The number of VRSA, VISA and hVISA isolates among different countries based on published case reports.

**Figure 8:**
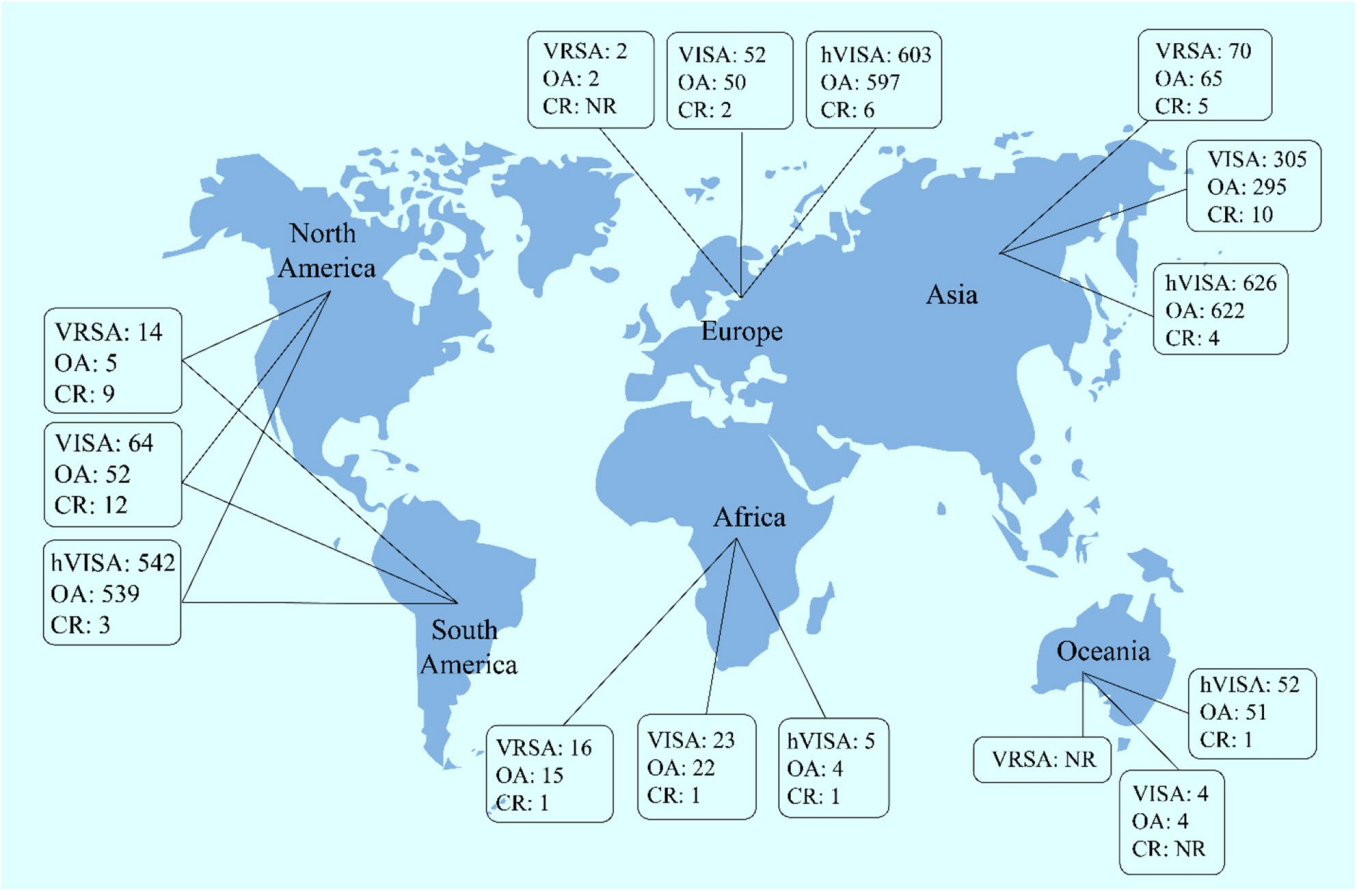
Number of VRSA, VISA and hVISA isolates in different continent based on published original and case report studies. *OA* original article, *CR* case report.

After the meta-analysis of the prevalence of VRSA, VISA and hVISA among human clinical isolates in different continents, we evaluated the frequency of these three types of *S. aureus* isolates based on case reports published in the mentioned electronic databases. Based on the results of case reports (Supplementry information Table S2) (which were not taken into account during the analyses already mentioned above), the numbers of VRSA, VISA and hVISA isolates were 12, 24 and 14 among different continents. However, most reports have been from Asia and America continents. There has been no report of VRSA isolates in Europe and Oceania. Oceania was the only continent from which there were no case reports on VISA (Figs. 7 and 8).

### Meta-regression analysis

The results of meta-regression showed that the prevalence of VISA was significantly increase by increasing published year (*p* value < 0.05, Supplementry information figure S1). The results of this analysis indicated that by increasing the published year of study, the prevalence of VRSA and hVISA increased, but this increase was statistically non-significant (*p* value > 0.05; Table 3 and Supplementry information Figure S1).

**Table 3:** Meta regression analysis of published year in prevalence VRSA, VISA and hVISA isolates among human clinical samples.

Moderator	Type of isolate	No studies	Coefficient	Standard error	*Z* value	*P* value
Published year	VRSA	23	0.03822	0.02051	1.86318	0.062
	VISA	50	0.10495	0.08376	9.70814	0.000
	hVISA	82	− 0.00016	0.00667	− 0.02334	0.981

## Discussion

Frequent use of vancomycin as the drug of choice for treatment of infections caused by multidrug-resistant MRSA has putatively led to selection of the is isolates with reduced susceptibility to vancomycin^15,203^. In this study we report the prevalence of VRSA, VISA and hVISA around the world. The global prevalence of VRSA, VISA and hVISA isolates was 1.5%, 1.7%, and 4.6%, respectively.

In studies mentioned in Supplementry information Table S1, presence of *vanA* in VRSA strains by PCR showed that 69% (55/79) of the VRSA strains were *vanA* positive. This elevated rate of *vanA* in these bacteria indicates that the resistance determinant was possibly acquired from a vancomycin-resistant *Enterococcus * species or from one of the other *vanA* positive organisms living in the human gastro-intestinal tract. Absence of *vanA* in the other isolates suggests that cell wall thickening and possibly vancomycin affinity trapping may be responsible for the development of vancomycin resistance in these isolates^19^. Furthermore, many studies reported a failure to detect the *vanB* gene.

Regarding VISA and hVISA strains, although, there is no clear overall genetic explanation for these phenotypes^15^, the main mechanisms of reduced susceptibility to vancomycin among VISA strains are mutations in cell wall-associated genes (thickened cell wall with an increased number of peptidoglycan layers)^204,205^, and/or in the ribosomal gene *rpoB*^203^. The prolonged usage of vancomycin can lead to changes in cell wall patterning or reduced expression of penicillin-binding proteins. This may accumulate from heterogeneous to selected homogeneous VISA-type resistance^203,206^. Noteworthy, when the cell wall gets thicker, the vancomycin MIC level increases^207,208^. Because of enhanced selective pressure, evolution of hVISA/VISA strains is more rapid in the hospital setting than in the community and therefore VISA is considered a more significant clinical problem than VRSA^209^. Furthermore, the results of meta-regression results showed the prevalence of the VISA was significantly increase over the time compare to the VRSA and hVISA. It seems that the real incidence of hVISA/ VISA strains is much higher than the present reports and hence there is a clear need for the development of new diagnostic methods for detecting hVISA/VISA. This also includes the development for new antimicrobial susceptibility tests (AST). For instance, in several studies, the PAP-AUC gold standard AST method was not used due to its time-consumption and technical difficulty. Other methods such as Disk Diffusion testing are unable to detect and distinguish these strains^9^. In overall, all studies used the culture-based methods such as E-test, PAP-AUC, broth dilution, and agar dilution. Moreover, some studies beside the culture-based methods, used PCR for detection of resistant-related gene. Since global scale sort of the same type and frequency of methods was used, the differences between developed and developing countries cannot be hypothetically addressed towards the use of different AST systems.

VRSA and/or VISA with resistance to multiple other antibiotics, including β-lactams, have been isolated from livestock animals that highlights the abuse of antibiotic in that sector and the suspected use of antibiotics as a food supplement^210,211^. The potential reasons for the emergence or detecting more resistant strains during recent years include: more frequent use of vancomycin for treatment of MRSA infections, better use of diagnostics, inadequate surveillance for drug-resistant strains and a possible change in the vancomycin-resistance breakpoints since 2006^212,213^.

The prevalence of VRSA in Asia, Europe, America and Africa was 1.2%, 1.1%, 3.6% and 2.5%, respectively. By the way, 65 strains of VRSA were found in Asia versus only 5 VRSA in America. The prevalence of VISA in Asia was higher than on the other continents. It should be noted that 67% (327/485) of vancomycin-resistant strains were reported from Iran and India. Therefore, our data shows that the Asian data are biased towards two countries but also that the emergence of VRSA in India and Iran warrants active microbiological surveillance and careful monitoring of vancomycin therapy. There are several factors involved in the higher number of VRSA and a higher prevalence of VISA in Asia, in comparison to Europe/America countries. Most of the Asian countries are developing countries with lower public hygiene standards and different attitudes towards antimicrobial treatments. Furthermore, population density can lead to more MRSA infections through enhanced microbial transmission. Higher vancomycin use for the treatment of infections can play a role as well^15^.

Previous studies have helped to identify risk factors that may contribute to VISA emergence such as previous MRSA colonization, hemodialysis dependence, long-term use of vancomycin, hospitalization in ICU and use of indwelling devices. There is no clarity on the precise clinical consequences of vancomycin non-susceptibility among *S. aureus* strains. Although some meta-analyses have addressed the association between elevated vancomycin MICs and worse clinical outcomes^214,215^, a recent prospective cohort study suggested exactly the opposite^216^. Previous studies have also demonstrated a correlation between increased vancomycin MICs and daptomycin resistance in VISA isolates^217,218^. Furthermore, decreased vancomycin susceptibility is associated with increased susceptibility to beta-lactams. Therefore, the combination of vancomycin and beta-lactams can be a good option for treatment of hVISA or VISA infections^198,219^. On the other hand, those correlated resistances can lead to problems in elucidating the role of the individual resistance marker in disease severity. Since there is an emerging and increasing rate of resistance to vancomycin, thorough monitoring of the success of vancomycin treatment is essential.^220^. The majority of VRSA strains belonged to same clonal complex (CC) such as CC5 in the USA. Interestingly, there is high prevalence of the CC5 in healthcare settings. Unlike VRSA, hVISA/VISA has been associated with many clones such as CC5, CC8, CC30, and CC45^221^.

Antibiotics such as trimethoprim-sulfamethoxazole, tetracyclines, fluoroquinolones, and clindamycin are alternative treatment choices for community-acquired MRSA infections. New drugs such as linezolid, daptomycin, tigecycline, and sodium fusidate are suggested for isolates with a vancomycin MIC of greater than 2 μg/mL^198^. Finally, in order to control the spread of vancomycin resistant staphylococci, contact precautions, disinfection of care equipment and the environment, plus adequate antimicrobial stewardship are highly recommended^222–224^.

## Supplementary information

Supplementary information
