# Supplementary material for: Global prevalence and distribution of vancomycin resistant, vancomycin intermediate and heterogeneously vancomycin intermediate Staphylococcus aureus clinical isolates: a systematic review and meta-analysis
Source: Sci Rep. 2020 Jul 29;10:12689. doi: 10.1038/s41598-020-69058-z (PMC7391782; doi:10.1038/s41598-020-69058-z)
Supplement: Supplementary file 1 — Supplementary information [file 41598_2020_69058_MOESM1_ESM.docx]

# Global Prevalence and Distribution of Vancomycin Resistant, Vancomycin Intermediate and Heterogeneously Vancomycin Intermediate *Staphylococcus aureus* Clinical Isolates: A Systematic Review and Meta-Analysis

**Aref Shariati^1,2^, Masoud Dadashi^3,4*^, Majid Taati Moghadam ^1,2^, Alex van Belkum^5^, Somayeh yaslianifard^3^, Davood Darban-Sarokhalil^1*^**

^1^Department of Microbiology, School of Medicine, Iran University of Medical Sciences, Tehran, Iran.

^2^Student Research Committee, Iran University of Medical Sciences, Tehran, Iran.

^3^Department of Microbiology, School of Medicine, Alborz University of Medical Sciences, Karaj, Iran.

^4^Non Communicable Diseases Research Center, Alborz University of Medical Sciences, Karaj, Iran.

^5^Open Innovation & Partnerships, Route de Port Michaud, 38390 La Balme Les Grottes, France.

**^*^Corresponding author(s):**

**Davood Darban-Sarokhalil, Ph.D.**

**Associate Professor**

Department of Microbiology, School of Medicine, Iran University of Medical Sciences, Tehran, Iran

Email Address**:** davood_darban@yahoo.com, darban.d@iums.ac.ir

Tel: +98 2186703183

Fax: +98 2188058649

**Masoud Dadashi, Ph.D.**

**Assistant Professor**

Department of Microbiology, School of Medicine, Alborz University of Medical Sciences, Karaj, Iran

Email Address: masoud.dadashi@sbmu.ac.ir, m_d6512@yahoo.com

Tel: +982634287339

Fax: +982634287311

**Table S1.** Characteristics of included studies which studied VRSA, VISA and hVISA isolates.

| First author | Time of study | Published time | Country | Total *S. aureus* | MRSA isolates | No of VRSA | No. of VISA isolates | No of hVISA isolate | Van A | Diagnostic methods for VRSA | Isolate source | Ward |
| --- | --- | --- | --- | --- | --- | --- | --- | --- | --- | --- | --- | --- |
| Bierbaum ^39^ | 1997 | 1999 | Germany | 307 | 307 |  | 2 |  |  | Brain heart infusion Agar plates | Different clinical sample |  |
| Ariza ^25^ | 1990-1997 | 1999 | USA | 19 | 19 |  |  | 14 |  | Agar dilution, PAP-AUC | Different clinical sample |  |
| Hubert ^96^ | 1997 | 1999 | USA | 630 | 630 |  | 4 |  |  | Agar dilution, PAP-AUC | Different clinical sample |  |
| Wong ^192^ | 1997-1998 | 2000 | Hong-Kong | 164 | 52 |  |  | 3 |  | E-test | Different clinical sample |  |
| Marchese ^121^ | 1997-1998 | 2000 | Italy | 179 | 179 | 2 |  |  |  | BMD | Different clinical sample |  |
| Trakulsomboon ^184^ | 1998-1999 | 2001 | Thailand | 155 | 155 |  |  | 3 |  | Agar dilution | Different clinical sample |  |
| Reverdy ^154^ | 1998-1999 | 2001 | France | 640 | 171 |  |  | 5 |  | Agar dilution, E-test | Different clinical sample |  |
| Oliveira ^141^ | 1998-1999 | 2001 | Brazil | 140 | 140 | 5 |  |  | 0 | Brain heart infusion Agar plates, BMD,E-test | Different clinical sample | Burn unit |
| Mi-Na Kim ^5^ | 1998-1999 | 2002 | Korea | 4483 | 3363 |  |  | 24 |  | E-test | Blood |  |
| Denis ^61^ | 1999 | 2002 | Belgium | 2145 | 881 |  | 3 | 4 |  | Agar dilution, E-test | Blood |  |
| Tallent ^178^ | 1997-2000 | 2002 | USA | 619 | 619 |  |  | 1 |  | Agar dilution | Blood |  |
| Russjan ^111^ | 2000-2001 | 2002 | Poland | 29 |  |  |  | 19 |  | E-test | Different clinical sample |  |
| Denis ^60^ | 1997-1998 | 2003 | Belgium | 434 | 384 |  | 11 |  |  | E-test, BMD | Different clinical sample |  |
| Bert ^36^ | 1997-2002 | 2003 | France | 48 | 48 |  |  | 13 |  | Agar dilution, E-test | Lower respiratory tract, Surgical Wound, Blood, The abdominal cavity |  |
| Mlynarczyk ^128^ | 2002 | 2003 | Poland | 103 | 103 |  |  | 5 |  | PAP-AUC | Different clinical sample |  |
| Lee ^113^ | 2001 | 2003 | Korea | 336 | 257 |  | 2 |  |  | Agar dilution | Different clinical sample |  |
| Charles ^13^ | 2001-2002 | 2004 | Australia | 53 | 53 |  |  | 5 |  | BMD, E-test | Blood |  |
| Song ^171^ | 1997-2000 | 2004 | India | 80 | 80 |  |  | 5 |  | Brain heart infusion Agar plates | Different clinical sample |  |
| Song ^171^ | 1997-2000 | 2004 | Japan | 231 | 231 |  |  | 19 |  | Brain heart infusion Agar plates | Different clinical sample |  |
| Song ^171^ | 1997-2000 | 2004 | Korea | 457 | 457 |  |  | 28 |  | Brain heart infusion Agar plates | Different clinical sample |  |
| Song ^171^ | 1997-2000 | 2004 | Philippines | 28 | 28 |  |  | 1 |  | Brain heart infusion Agar plates | Different clinical sample |  |
| Song ^171^ | 1997-2000 | 2004 | Singapore | 87 | 87 |  |  | 2 |  | Brain heart infusion Agar plates | Different clinical sample |  |
| Song ^171^ | 1997-2000 | 2004 | Thailand | 96 | 96 |  |  | 2 |  | Brain heart infusion Agar plates | Different clinical sample |  |
| Song ^171^ | 1997-2000 | 2004 | Vietnam | 41 | 41 |  |  | 1 |  | Brain heart infusion Agar plates | Different clinical sample |  |
| Cartolano^43^ | 2000 | 2004 | France | 1070 | 1070 |  | 31 |  |  | Agar dilution, E-test | Different clinical sample |  |
| Pierard ^150^ | 2003 | 2004 | Belgium | 1002 | 1002 |  | 1 | 2 |  | Agar dilution, PAP-AUC | Different clinical sample |  |
| Khosrovaneh ^103^ | 2002 | 2004 | USA | 22 | 22 |  |  | 3 |  | E-test | Blood |  |
| Sancak ^163^ | 1998-2002 | 2005 | Turkey | 256 | 256 |  |  | 46 |  | E-test | Different clinical sample |  |
| Nonhoff ^136^ | 2001 | 2005 | Belgium | 455 | 455 |  |  | 3 |  | E-test | Different clinical sample |  |
| Melo ^123^ | 2000-2002 | 2005 | Brazil | 41 | 32 |  | 1 | 8 |  | Agar dilution | Swabing oral and rectal cavities |  |
| Bataineh ^34^ | 2002-2004 | 2006 | Jordan | 126 |  | 5 |  |  |  | E-test | Different clinical sample |  |
| Robert ^158^ | 1983-2001 | 2006 | France | 1445 | 1445 |  | 1 |  |  | E-test | Different clinical sample |  |
| Tiwari ^182^ | 2002-2005 | 2006 | India | 783 | 318 | 2 | 6 |  | 0 | Brain heart infusion Agar plates | Pus, Urine, Wound swabs, Catheters, Blood, Sputum and CSF |  |
| Garnier ^76^ | 2001-2002 | 2006 | France | 2300 | * |  |  | 255 |  | Brain heart infusion Agar plates | Different clinical sample |  |
| Hakim ^84^ | 2006-2007 | 2007 | Pakistan | 850 | 250 |  | 32 |  |  | E-test | purulent drainage, Wound swabs, Urine, Ear swabs and Blood |  |
| Hanaki ^87^ | 1978-2005 | 2007 | Japan | 2446 | 2446 |  | 8 |  |  | E-test | Different clinical sample |  |
| Maor ^120^ | 2003-2004 | 2007 | Israel | 264 | 264 |  |  | 16 |  | E-test | Blood |  |
| Neoh ^135^ | 1998-2005 | 2007 | Japan | 20 | 20 |  | 2 | 2 |  | PAP-AUC | Blood |  |
| Fitzgibbon ^71^ | 1998-2004 | 2007 | Ireland | 3189 | 3189 |  |  | 79 | * | PAP-AUC | Different clinical sample |  |
| Aligholi ^19^ | 2005 | 2008 | Iran | 356 | 149 | 2 |  |  | 1 | BMD | Different clinical sample |  |
| Chua ^53^ | 2005-2007 | 2008 | USA | 210 | 210 |  |  | 30 |  | E-test | Blood |  |
| Rybak ^160^ | 1986-2007 | 2008 | USA | 1499 | 1499 |  | 12 | 108 |  | E-test, GRD | Blood, lung, skin |  |
| Shick ^110^ | 2006-2007 | 2008 | USA | 982 | 982 |  | 3 | 2 |  | E-test | Different clinical sample |  |
| Fong ^72^ | 2005-2006 | 2009 | Singapore | 56 | 56 |  | 7 | 3 |  | E-test | Blood |  |
| Sader ^161^ | 2002-2006 | 2009 | USA | 268 | 268 |  |  | 36 |  | Macro method E-test | Blood |  |
| Sun ^174^ | 2005-2007 | 2009 | China | 200 | 200 |  | 1 | 26 |  | E-test | Blood |  |
| Lulitanond ^118^ | 2002-2003, 2006-2007 | 2009 | Thailand | 894 | 894 |  | 3 | 12 |  | Brain heart infusion Agar plates, PAP-AUC | Different clinical sample |  |
| Musta ^131^ | 1996-2006 | 2009 | USA | 489 | 489 |  |  | 71 |  | BMD, vancomycin-teicoplanin E-test (macro method) | Blood |  |
| Horne ^92^ | 2005 | 2009 | Australia | 117 | 117 |  | 2 | 56 |  | PAP-AUC, BMD | Different clinical sample |  |
| Cafiso ^41^ | 2005-2007 | 2010 | Italy | 64 | 14 |  |  | 7 |  | BMD, E-test | Cystic fibrosis |  |
| Campanile ^42^ | 2005-2007 | 2010 | Italy | 1284 | 1284 |  |  | 36 |  | Agar dilution, E-test | Blood stream, Pneumonia and Skin-structure infections |  |
| Adam ^18^ | 1995-2006 | 2010 | Canada | 475 | 475 |  |  | 25 |  | E-test, BMD | Different clinical sample |  |
| Ho ^90^ | 2003 | 2010 | Taiwan | 1000 | 1000 |  | 2 | 7 |  | Agar dilution, PAP-AUC | Different clinical sample |  |
| Hsueh ^93^ | 2001-2002 | 2010 | Taiwan | 1500 | 1500 |  | 43 |  |  | BMD | Different clinical sample |  |
| Chung ^56^ | 2001-2006 | 2010 | Korea | 41639 | 41639 |  | 15 | 18 |  | Agar dilution, BMD | Different clinical sample |  |
| Chung ^55^ | 2001-2006 | 2010 | Korea | 37856 | 37856 |  | 33 | 18 |  | BMD | Different clinical sample |  |
| Kirby ^108^ | 2006-2006 | 2010 | UK | 2550 | 2550 |  |  | 86 |  | Macro method E-test | Different clinical sample |  |
| Monaco ^129^ | 2006-2007 | 2010 | Italy | 148 | 66 |  | 2 | 9 |  | E-test | Different clinical sample |  |
| Ouko ^144^ |  | 2010 | Kenya | 118 | 31 |  | 5 |  |  | E-test | Different clinical sample |  |
| Cha ^45^ | 2001-2007 | 2010 | Korea | 448 | 448 |  | 4 |  |  | Agar dilution, PAP-AUC | Different clinical sample |  |
| Gallon ^74^ | 2007 | 2010 | France | 166 | 51 |  | 2 |  |  | E-test, PAP-AUC | Blood |  |
| Chen ^12^ | 2005-2008 | 2011 | China | 559 | 559 |  |  | 62 |  | Agar dilution, PAP-AUC | Different clinical sample |  |
| Goud ^79^ | 2003-2007 | 2011 | India | 282 | 205 | 4 |  |  | 4 | PCR for van | Swab from anterior nares, forearm, dorsum and palm of the hands |  |
| Khatib ^101^ | 2002-2003,2005-2006 | 2011 | USA | 371 | 371 |  | 6 | 30 |  | BMD, E-test | Blood |  |
| Richter ^156^ | 2009 | 2011 | USA | 4210 | 2248 |  |  | 11 |  | E-test, GRD | Different clinical sample |  |
| Riederer ^157^ | 1996-2006 | 2011 | USA | 485 | 485 |  | 7 | 33 |  | PAP-AUC | Blood |  |
| Thati ^180^ | 2008 | 2011 | India | 358 | 285 | 7 | 16 |  | 6 | Agar dilution, PCR for VAN | Blood, Urine and throat swabs, Wound and ear swabs |  |
| van Hal ^187^ | 1996-2008 | 2011 | Australia | 401 | 401 |  | 2 | 46 |  | PAP-AUC | Blood |  |
| Rebiahi ^153^ | 2007-2009 | 2011 | Algeria | 220 | 165 | 3 |  |  |  | Dilution method on solid medium | Wound |  |
| Pitz ^151^ | 2000-2008 | 2011 | USA | 167 | 167 |  |  | 2 |  | E-test, Microscan, PAP-AUC | Blood |  |
| Alzolibani ^21^ | 2009-2010 | 2012 | Saudi Arabia | 80 | 29 | 9 |  |  |  | Vitek | skin |  |
| Gagliotti ^73^ | 2009 | 2012 | Italy | 267 | 106 |  |  | 27 |  | E-test, BMD | Blood, Respiratory tract, Wound |  |
| Ahmad ^137^ | 2009 | 2012 | Malaysia | 43 | 43 |  |  | 2 |  | E-test | Different clinical sample |  |
| Anvari ^24^ | 2011 | 2012 | Iran | 54 | 32 | 3 |  |  | 3 | BHI vancomycin screen Agar | Wound swab |  |
| Havaei ^89^ | 2011 | 2012 | Iran | 171 | 115 |  | 5 |  |  | Agar dilution, E-test | Blood, Urine, Sputum, Wound, Abscess, Nose, Throat, Eye, and Respiratory tract |  |
| Banerjee ^33^ | 2010 | 2012 | India | 135 | 135 |  | 4 |  |  | Agar dilution, BMD | Nasal swab | ICU |
| Ramli ^152^ | 2009 | 2012 | Malaysia | 320 | 320 |  |  | 7 |  | E-test, GRD | Different clinical sample |  |
| Yamakawa ^193^ | 1990 | 2012 | Japan | 750 | 750 |  |  | 38 |  | Brain heart infusion Agar plates | Different clinical sample |  |
| Lin ^116^ | 2009 | 2012 | Taiwan | 118 | 62 |  |  | 4 |  | E-test | Blood |  |
| Park ^147^ | 2008-2010 | 2012 | Korea | 268 | 268 |  |  | 101 |  | E-test | Blood |  |
| Parer ^146^ | 2007 | 2012 | France | 20 | 20 |  |  | 12 |  | E-test, PAP-AUC | Different clinical sample | ICU |
| Hafer ^81^ | 2007-2008 | 2012 | USA | 77 | 77 |  | 22 | 9 |  | Microscan-test, PAP-AUC | Different clinical sample |  |
| Gowrishankar ^80^ | 2009-2010 | 2013 | India | 165 | 63 |  | 10 |  |  | BMD | Throat swabs |  |
| Jian Hu ^94^ | 2007-2011 | 2013 | China | 757 | 369 |  | 4 | 76 |  | BMD | Sputum, Pus, Blood, Wound |  |
| Dubey ^64^ | 2009-2015 | 2013 | India | 1507 | 1214 | 251 | 545 |  |  | BMD | Different clinical sample | ICU, NICU |
| Sancak ^164^ | 2009-2010 | 2013 | Turkey | 175 | 175 |  |  | 24 |  | E-test, PAP-AUC | Different clinical sample |  |
| Takata ^177^ | 1987-2007 | 2013 | Japan | 162 | 162 |  |  | 30 |  | Macro method E-test | Blood |  |
| Wang ^190^ | 2005-2009 | 2013 | Taiwan | 284 | 284 |  |  | 16 |  | E-test, GRD | Blood |  |
| Wang ^190^ | 2007-2009 | 2013 | China | 122 | 122 |  |  | 25 |  | E-test | Different clinical sample |  |
| Chaudhary ^50^ | 2013 | 2013 | India | 413 | 211 |  |  | 8 |  | Agar dilution, E-test | Pus, Blood, Urine, Wound swab and ear swab samples |  |
| Oksuz ^140^ | 2007-2012 | 2013 | Turkish | 102 | 102 |  |  | 3 |  | E-test | Different clinical sample |  |
| Muneeri ^130^ | 2011-2012 | 2013 | Iran | 73 |  | 1 | 11 |  | 1 | E-test. | Different clinical sample |  |
| Hanaki ^86^ | 2008-2011 | 2014 | Japan | 830 | 830 |  | 8 | 54 |  | BMD | Blood |  |
| Richter ^155^ | 2011 | 2014 | USA | 4131 | 2093 |  |  | 47 |  | E-test, GRD | Wounds or abscesses, Blood, Lower respiratory tract , Tissue, and other normally sterile sites |  |
| Yoon ^194^ | 2012 | 2014 | Korea | 103 | 103 |  |  | 3 |  | PAP-AUC | Pus, Biopsy, Wound, Body fluid, and Sputum |  |
| Panomket ^181^ | 2010-2011 | 2014 | Thailand | 68 | 68 |  |  | 2 |  | BMD | Different clinical sample |  |
| El Ayoubi ^65^ | 2006-2013 | 2014 | Lebanon | 113 | 113 |  |  | 5 |  | MIC based | Different clinical sample |  |
| Casapao ^44^ | 2002-2013 | 2014 | USA | 202 | 202 |  | 3 | 38 |  | BMD,PAP-AUC | Blood |  |
| Silveira ^166^ | 2009-2013 | 2014 | Brazil | 124 | * |  |  | 12 |  | E-test, PAP-AUC | Different clinical sample |  |
| Islam ^97^ | 2011-2012 | 2015 | Bangladesh | 44 | 15 | 2 |  |  | 1 | Agar dilution, BMD | Wound swab |  |
| Chung ^54^ | 2004-2006 | 2015 | South Korea | 114 | 114 |  |  | 8 |  | E-test | Blood, Sputum, Pus  Urine, Ascites, Pleural Fluid, Catheter tip |  |
| Chung ^54^ | 2004-2006 | 2015 | Taiwan | 104 | 104 |  |  | 2 |  | E-test | Pus, Blood |  |
| Chung ^54^ | 2004-2006 | 2015 | Vietnam | 71 | 71 |  |  | 5 |  | E-test | Pus, Blood, Sputum |  |
| Chung ^54^ | 2004-2006 | 2015 | Thailand | 31 | 31 |  |  | 1 |  | E-test | Urine |  |
| Khatib ^102^ | 2002-2003, 2005-2006,2008-2009, 2010-2012 | 2015 | USA | 720 | 720 |  | 10 | 37 |  | BMD, E-test | Blood |  |
| Chaudhary ^51^ | 2013-2014 | 2015 | India | 165 | 165 | 6 | 21 |  | 6 | BHI vancomycin screen agar | Blood, Urine, Pus, Throat swabs, Wound, Ear swabs |  |
| Di Gregorio ^63^ | 2009-2010 | 2015 | Argentina | 92 | 44 |  |  | 3 |  | E-test | Blood |  |
| Chaudhari ^49^ | 2010-2013 | 2015 | India | 58 | 58 |  |  | 4 |  | E-test, Agar dilution | Different clinical sample |  |
| Singh ^167^ | 2011-2014 | 2015 | India | 500 | 500 |  |  | 29 |  | E-test, Agar dilution | Different clinical sample |  |
| Liu ^117^ | 2011-2012 | 2015 | China | 184 | 77 |  |  | 17 |  | Agar dilution, PAP-AUC | Blood, CSF, Ascites, Pleural effusion, Synovial fluid |  |
| Mirza ^127^ | 2001-2011 | 2015 | Turkish | 94 | 94 |  |  | 20 |  | BMD, E-test | Different clinical sample |  |
| Park ^148^ | 2012-2013 | 2015 | Korea | 118 | 118 |  |  | 4 |  | E-test | Blood |  |
| Liaqat ^114^ |  | 2015 | Pakistan | 150 | 51 | 5 | 22 |  |  | BMD | Different clinical sample |  |
| Kang ^100^ | 2006-2007, 2011-2013 | 2016 | Korea | 372 | 372 |  |  | 22 |  | BMD, E-test | Different clinical sample |  |
| Claeys ^57^ | 2005-2014 | 2016 | USA | 87 |  |  |  | 29 |  | BMD | Pneumonia |  |
| Abdel-Maksoud ^16^ | 2005-2013 | 2016 | Egypt | 631 | 343 |  | 4 |  |  | BMD | Pus, Wound swabs ,Urine, Blood, Bronchoalveolar lavage, endotracheal tube, Sputum |  |
| Hasan ^88^ | 2012-2013 | 2016 | Bangladesh | 40 | 21 | 8 |  |  |  | BMD | Burn Wound |  |
| Huang ^95^ | 2012-2013 | 2016 | Taiwan | 622 | 622 |  | 17 | 2 |  | E-test | Blood, CSF, Ascites, and Pleural effusion |  |
| da Costa ^58^ | 2011-2013 | 2016 | Brazil | 110 | 31 |  | 6 | 1 |  | BMD, E-test | Blood |  |
| Ullah ^186^ | 2012-2013 | 2016 | Pakistan | 280 | 101 |  | 7 |  |  | E-test | Different clinical sample |  |
| Rae Koh ^109^ | 2012-2013 | 2016 | Korea | 229 | 138 |  |  | 79 |  | E-test | Blood isolates, Body fluids, Bronchial fluid, Pus, Sputum Urine |  |
| Mendem ^125^ |  | 2016 | India | 212 | 100 | 23 |  |  |  | Agar dilution | Different clinical sample |  |
| Peerayeh ^133^ | 2009-2012 | 2016 | Iran | 414 |  |  | 10 |  |  | E-test | Different clinical sample |  |
| Neetu ^134^ | 2010-2014 | 2016 | India | 259 | 259 |  | 10 |  |  | E-test | Different clinical sample |  |
| Kim ^104^ | 2009-2011 | 2016 | Korea | 1717 | 1066 |  |  | 42 |  | PAP-AUC | sterile body fluid |  |
| Kumar ^112^ | 2013-2015 | 2016 | India | 47 | 28 | 2 |  |  | 2 | Vitek 2 Compact automated system | Blood |  |
| Daghistani ^85^ | * | 2017 | Jordan | 566 | * |  | 4 |  |  | E-test, Agar dilution | Skin, Nasal, Nail and other |  |
| Gecgel ^77^ | 2011-2015 | 2017 | Turkey | 73 | 23 |  | 2 | 41 |  | Microscan (Walkaway, Beckman Coulter, USA) | Blood |  |
| Fasihi ^68^ | 2014-2015 | 2017 | Iran | 170 | 90 | 2 |  |  | 1 | Agar dilution, BMD | Urine, Wound, CSF, Bronchoalveolar lavage |  |
| Yousefi ^195^ | 2014-2015 | 2017 | Iran | 54 | 30 | 2 |  |  | 2 | BMD | UTI |  |
| Shekarabi ^165^ | 2014-2017 | 2017 | Iran | 1789 | * | 4 | 2 |  | 1 | E-test | Wound, Blood, Pus, Urine, Catheters and Body fluids |  |
| Vellappally ^189^ |  | 2017 | Saudi Arabia | 150 | 98 | 15 | 27 |  | 13 | Agar dilution | Dental caries |  |
| Olufunmiso ^142^ | 2015-2016 | 2017 | Nigeria | 200 |  | 89 | 30 |  |  | Agar dilution | Different clinical sample |  |
| T. Kim ^106^ | 2008-2010 | 2017 | Korea | 235 | 235 |  |  | 24 |  | BMD | Blood |  |
| Martirosov ^122^ | 2005-2009 | 2017 | USA | 238 | 119 |  |  | 7 |  | BMD | Blood |  |
| Bamigboye ^32^ | 2015-2016 | 2018 | Nigeria | 73 | 5 | 1 | 11 |  | 0 | E-test | Different clinical sample |  |
| Fasihi ^69^ | 2015-2016 | 2018 | Iran | 205 | 100 | 2 |  |  | 2 | Agar dilution, BMD | Urine, Wound, CSF, Bronchoalveolar lavage |  |
| Ghahremani ^78^ | 2012-2015 | 2018 | Iran | 177 | 95 | 11 | 1 |  | 9 | BHI vancomycin screen agar, E-test | Different clinical sample |  |
| Jahanshahi ^98^ | 2015-2016 | 2018 | Iran | 100 | 46 | 2 | 4 |  | 2 | Agar dilution | Blood, Sputum, Wound swabs, Chest tube secretion, Urine |  |
| AbdEl-Mongy ^17^ | * | 2018 | Egypt | 50 | 50 |  | 1 |  |  | Agar dilution | Blood | NICU |
| ElSayed ^67^ | 2013-2014 | 2018 | Egypt | 200 | 90 | 11 | 1 |  |  | Agar dilution, E-test | Abscesses, diabetic foot infections, Postoperative Wound infections, and skin infections |  |
| Abd El-Aziz ^66^ | * | 2018 | Egypt | 17 | 15 | 3 |  |  | 0 | BMD | Pus, Urine, Sputum, CSF, Blood, Peritoneal fluid, Pericardial fluid |  |
| Asadpour ^26^ | 2017 | 2019 | Iran | 110 | 47 | 3 | 8 |  | 1 | BMD | Different clinical sample |  |
| Bakthavatchalam ^30^ | 2016-2017 | 2019 | India | 50 | 10 |  |  | 29 |  | BMD | Blood |  |
| Othman ^143^ | 2017 | 2019 | Egypt | 100 | 100 |  | 22 | 9 |  | BMD | Different clinical sample |  |

BMD; Broth Microdilution. ICU: Intensive Care Unit. NICU: Neonatal, Intensive Care Unit. BHI: Brain Heart Infusion. UTI: Urinary Tract Infection. PAP-AUC: Population Analysis Profile/Area Under the Curve. GRD: Glycopeptide Resistance Detection. MIC: Minimum Inhibitory Concentration.

**Table S2**. Detailed information available for VRSA, VISA and hVISA cases report.

| Author | Publish time | country | Patient | Isolate | Detection method | Susceptible antibiotics | Resistant antibiotics |
| --- | --- | --- | --- | --- | --- | --- | --- |
| Backo ^29^ | 1999 | USA | Endocarditis | VISA | BMD |  |  |
| Wong ^192^ | 2000 | Hong-Kong | Four different cases with bacteremia | VRSA | E-test |  |  |
| KIM ^105^ | 2000 | Korea | A 45-year-old man with sigmoid colon cancer | VISA | Serial diluents onto BHI agar plates | Rifampin, cotrimoxazole, and vancomycin | Ciprofloxacin, clindamycin, erythromycin, gentamicin, and tetracycline |
| Bobin-Dubreux ^40^ | 2001 | France | 35-year-old woman with conjunctivitis | hVISA | E-test, BMD |  |  |
| Hageman ^83^ | 2001 | USA | 27-year-old home health-care patient following a complicated cholecystectomy. | VISA | BMD |  |  |
| Tsakris ^185^ | 2002 | Greece | 52-year-old man with traffic accident | VISA | Agar dilution, BMD, E-test | Chloramphenicol, cotrimoxazole, fosfomycin, gentamicin, kanamycin, nitrofurantoin, ofloxacin | Tobramycin, macrolides, tetracyclines, rifampicin, fusidic acid |
| Zeller ^196^ | 2002 | France | A 70-y-old otherwise healthy male with Hip arthroplasty infection | hVISA | PAP-AUC, E-test |  |  |
| Andrade-Baiocchi ^23^ | 2003 | Brazil | 52-year-old woman with Endocarditis | VISA | E-test |  |  |
| Whitener ^191^ | 2003 | USA | A 70-year-old with an infected chronic heel ulcer | VRSA | BMD | Minocycline, Gatifloxacin | Oxacillin, Tetracycline, Levofloxacin, Gentamicin, Tobramycin, Amikacin, Chloramphenicol, Erythromycin |
| Chang ^48^ | 2003 | USA | a 40-year-old woman with foot ulcers infection | VRSA | MIC based |  |  |
| Naimi ^132^ | 2003 | USA | 59-year-old man with diabetes, hypertension, atrial fibrillation, peripheral vascular disease, and hepatitis C | VISA | BMD, E-test |  |  |
| Amod ^22^ | 2004 | South Africa | A 67-year mate with ventriculitis. | hVISA | E-test |  |  |
| Sng ^169^ | 2005 | Singapore | 74-year old man with cancer of the esophagus and post-surgical infection, A 68-year old man with chronic renal failure and MRSA sepsis | hVISA | E-test macro-method |  |  |
| Mabed ^119^ | 2005 | Egypt | 38-year-old lady with Hodgkin’s disease | VISA | Broth microdilution methods | Tetracycline and chloramphenicol | Erythromycin, clindamycin, trimethoprim-sulfamethoxazole, and ciprofloxacin. |
| Phongsamart ^149^ | 2005 | Thailand | 4 month-old girl with truncus arteriosus type IV and ventricular septal defect | hVISA | E-test | Co-trimoxazole |  |
| Finks ^70^ | 2007 | USA | 48-year-old patient with right plantar foot wound, a 54-year-old patient 54-year-old patient with left plantar foot wound | VRSA | MIC based | Linezolid, meropenem and daptomycin |  |
| Julian ^99^ | 2007 | USA | One patient with a prosthetic aortic valve who, as a complication of a bacteremic pacemaker infection | VISA | BMD |  | Daptomycin |
| Hong ^91^ | 2008 | Korea | 59 year -old man undergone total hip replacement arthroplasty, 57 year -old man with aspiration pneumonia | two VISA | BMD |  | Penicillin, erythromycin, clindamycin, and gentamicin |
| Gardete ^75^ | 2008 | Portugal | Female patient with surgical in hip | VISA | PAP-AUC, E-test | Clindamycin, gentamicin, rifampin, tetracycline, trimethoprim=sulfamethoxazole, and linezolid | Ciprofloxacin |
| Hageman ^82^ | 2008 | USA | 46-year-old patient with endocarditis | VISA | BMD |  |  |
| Chaiwongkarjohn ^46^ | 2009 | Taiwan | 67-year-old female and 19-year-old male with Septic Arthritis | two VISA | E-test |  |  |
| Mirani ^126^ | 2009 | Pakistan | In-patient of local Cardiac Hospital of Karachi | VISA | E-test |  |  |
| Balkhair ^31^ | 2010 | Oman | A 57-year-old male with endocarditis | VISA | E-test | Linezolid |  |
| Al-Obeid ^20^ | 2010 | Saudi Arabia | 69-year-old Saudi male patient with severe sepsis | hVISA | E-test, BMD, PAP-AUC |  |  |
| Sola ^170^ | 2011 | Argentina | A 73-year-old female with Endocarditis | hVISA | MHA5T-screening agar, Macro method E-test-(MET), GRD E-test |  |  |
| Tascini ^179^ | 2011 | Italy | A 72-year-old man with bacteremia | hVISA | E-test |  |  |
| Chaiwongkarjohn ^47^ | 2011 | USA | A 61-year-old Japanese woman with Bacteremia | VISA | E-test, BMD |  |  |
| Azimian ^28^ | 2012 | Iran | 26-year-old man with ileal perforation and peritonitis. | VRSA | E-test | Minocycline, and gentamicin. | Levofloxacin, ciprofloxacin, tetracycline, cotrimoxazole, clindamycin, and rifampin |
| Dezfulian ^62^ | 2012 | Iran | A 51-year-old female with Diabetic Foot Ulcer | VRSA | BMD | Imepenem | penicillin, oxacillin, ceftriaxone, erythromycin, clindamycin, amikacin, co-trimoxazole, chloramphenicol, amoxicillin |
| Sivakumar ^168^ | 2012 | Australia | An 82-year-old man undergoing selective left total knee arthroplasty | hVISA | PAP-AUC |  | Teicoplanin, daptomycin |
| Stroh ^172^ | 2012 | USA | An 83-yearold man with endophthalmitis | VRSA | Vitek 2 system; BioMérieux | Chloramphenicol, quinupristin/dalfopristin, and linezolid | Methicillin, vancomycin, moxifloxacin, clindamycin, imipenem, and tetracycline |
| Avery ^27^ | 2012 | USA | An 80-year-old male with Osteomyelitis and bacteremia | VISA | BMD |  | Daptomycin |
| Panesso ^145^ | 2012 | Brazil | Blood | VRSA | . |  |  |
| Beydoun ^37^ | 2013 | USA | A 37-year-old African American male with Endocarditis | VISA | MIC based | Clindamycin, Gentamicin, Tetracycline, Trimethoprim/sulfamethoxazole | Ciprofloxacin, Daptomycin,Erythromycin, Levofloxacin, Linezolid, Moxifloxacin, Oxacillin, Rifampin |
| Swartz ^175^ | 2013 | USA | a 44-year-old woman with idiopathic dilated cardiomyopathy | hVISA | Double-headed E-tests |  |  |
| Zhang ^197^ | 2013 | China | 34-year-old male patient with steam burn | VISA | E-test |  |  |
| Chen ^52^ | 2013 | Taiwan | 67-year-old with sepsis woman | VISA | E-test |  |  |
| Wirtz ^124^ | 2013 | USA | A 74-year-old woman with diabetes mellitus, chronic renal failure requiring hemodialysis, and peripheral vascular disease | VRSA | Vitek, Micro Scan systems both | Co-trimoxazole | Erythromycin, Clindamycin, |
| Rossi ^159^ | 2014 | Brazil | 35-year-old man with a bloodstream infection | VRSA | Agar dilution, broth microdilution |  |  |
| Limbago ^115^ | 2014 | USA | 70-year-old man with hypertension and diabetes | VRSA | BMD |  | Cefoxitin, vancomycin, clindamycin, erythromycin, levofloxacin, and tetracycline; intermediately resistant to doxycycline and minocycline; and susceptible to chloramphenicol, daptomycin, gentamicin, linezolid, rifampin, tigecycline, and trimethoprim- sulfamethoxazole |
| Kino ^107^ | 2014 | Japan | A 77-year-old with decreased consciousness level and vomiting | VISA | Microscan panel, E-test | Macrolides, clindamycin, minocycline, and fluoroquinolones | Macrolides, clindamycin, minocycline, and fluoroquinolones. |
| Baxi ^35^ | 2015 | USA | A 81-year old woman with Endocarditis | VISA | BMD |  |  |
| Dedania ^59^ | 2015 | USA | A 48-year-old woman with Endophthalmitis | VRSA | MIC based |  |  |
| Zhu ^198^ | 2015 | China | 51-year-old man with a complaint of dysphagia for | hVISA | Vitek 2 compact automated system (bioMérieux, Marcy-l’E´ toile, France), PAP-AUC |  |  |
| Gregorio ^226^ | 2015 | Argentina | A 30-year-old man suffering M4 acute myeloid leukemia | hVISA | BMD |  |  |
| Sambandam ^162^ | 2015 | India | Three case with Intramuscular Injection Abscess | VRSA | . |  |  |
| Varona-Barquín ^188^ | 2016 | Spain | Five-year-old girls with tetralogy of Fallot and cardiovascular surgery. | hVISA | Macro E-test |  |  |
| Tóth ^183^ | 2016 | Hungary | 47-year-old man with B-type aortic dissection | hVISA | E-test | Linezolid, daptomycin | Ciprofloxacin, gentamicin, erythromycin, clindamycin |
| Oguz ^138^ | 2017 | Turkey | A 37-year old male with orthopedic implant after a traffic accident | hVISA | E-test, PAP-AUC |  |  |
| Okada ^139^ | 2018 | Japan | 66-year old male with enteritis | VRSA | . |  |  |
| Bhowmick ^38^ | 2019 | USA | 7 different cases with bacteremia | VISA | E-test, BMD |  |  |
| Taha ^176^ | 2019 | Egypt | A 50-year-old comatose woman with hospital-acquired pneumonia | VRSA | MIC based | Linezolid | Penicillin, amoxicillin/clavulanic, ampicillin/ sulbactam, cefazolin, cefuroxime, gentamicin and ciprofloxacin |
| Sumon ^173^ | 2019 | USA | A 73-year-old Caucasian male with prosthetic aortic valve endocarditis | VISA | MIC based |  | Daptomycin |

BMD; Broth Microdilution. MIC: Minimum Inhibitory Concentration. BHI: Brain Heart Infusion. UTI: Urinary Tract Infection. PAP-AUC: Population Analysis Profile/Area Under the Curve. GRD: Glycopeptide Resistance Detection.


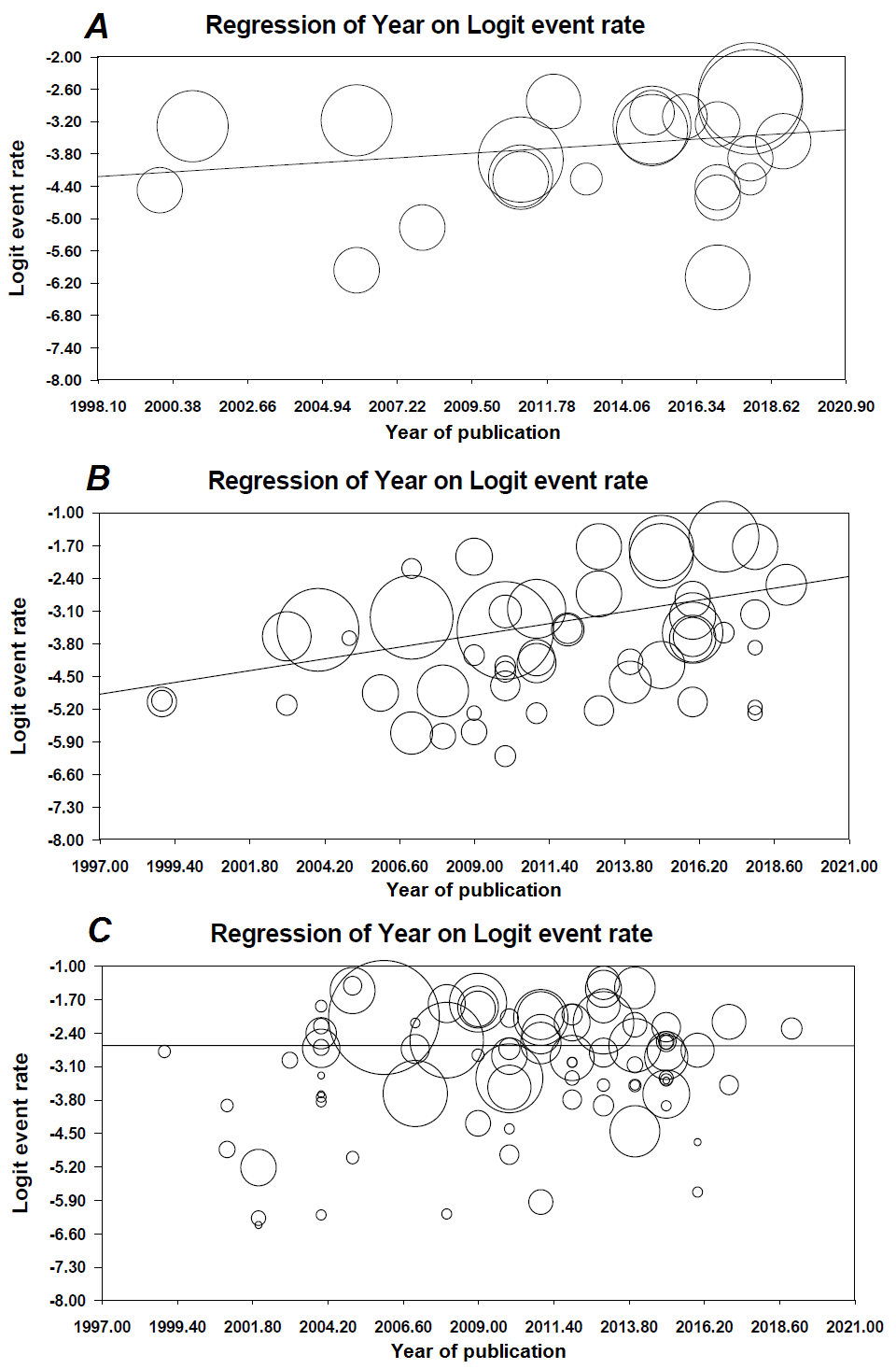


Figure S1. Meta-regression plots of prevalence of (A) VRSA, (B) VISA and (C) hVISA isolates in human clinical samples using the published year as the moderator.

**References**

1 Abbasian, S. *et al.* Genotypic characterization of Staphylococcus aureus isolated from a burn centre by using agr, spa and SCCmec typing methods. *New microbes and new infections* **26**, 15-19 (2018).

2 Bamigboye, B. T., Olowe, O. A. & Taiwo, S. S. Phenotypic and molecular identification of vancomycin resistance in clinical Staphylococcus aureus isolates in Osogbo, Nigeria. *European Journal of Microbiology and Immunology* **8**, 25-30 (2018).

3 Shanson, D., Kensit, J. & Duke, R. Outbreak of hospital infection with a strain of Staphylococcus aureus resistant to gentamicin and methicillin. *The Lancet* **308**, 1347-1348 (1976).

4 HALEY, R. W. *et al.* The emergence of methicillin-resistant Staphylococcus aureus infections in United States hospitals: possible role of the house staff-patient transfer circuit. *Annals of Internal Medicine* **97**, 297-308 (1982).

5 Kim, M.-N., Hwang, S. H., Pyo, Y.-J., Mun, H.-M. & Pai, C. H. Clonal spread of Staphylococcus aureus heterogeneously resistant to vancomycin in a university hospital in Korea. *Journal of clinical microbiology* **40**, 1376-1380 (2002).

6 Hiramatsu, K. *et al.* Methicillin-resistant Staphylococcus aureus clinical strain with reduced vancomycin susceptibility. *The Journal of antimicrobial chemotherapy* **40**, 135-136 (1997).

7 Goldrick, B. First Reported Case of VRSA in the United States: An alarming development in microbial resistance. *AJN The American Journal of Nursing* **102**, 17 (2002).

8 Francia, M. V. & Clewell, D. B. Transfer origins in the conjugative Enterococcus faecalis plasmids pAD1 and pAM373: identification of the pAD1 nic site, a specific relaxase and a possible TraG‐like protein. *Molecular microbiology* **45**, 375-395 (2002).

9 Tenover, F. C., Biddle, J. W. & Lancaster, M. V. Increasing resistance to vancomycin and other glycopeptides in Staphylococcus aureus. *Emerging infectious diseases* **7**, 327 (2001).

10 Woodford, N. Epidemiology of the genetic elements responsible for acquired glycopeptide resistance in enterococci. *Microbial Drug Resistance* **7**, 229-236 (2001).

11 AL-DAGHISTANI, H. I., D SHQUIRAT, W., Al-Kharabsha, M. & AL-LATIF, S. M. A. Asymptomatic colonization of Staphylococcus aureus with intermediate resistance to vancomycin harboring vanb resistance gene. *Asian J. Pharm. Clin. Res.* **10**, 349-356 (2017).

12 Chen, H., Liu, Y., Sun, W., Chen, M. & Wang, H. The incidence of heterogeneous vancomycin-intermediate Staphylococcus aureus correlated with increase of vancomycin MIC. *Diagnostic microbiology and infectious disease* **71**, 301-303 (2011).

13 Charles, P. G., Ward, P. B., Johnson, P. D., Howden, B. P. & Grayson, M. L. Clinical features associated with bacteremia due to heterogeneous vancomycin-intermediate Staphylococcus aureus. *Clinical infectious diseases* **38**, 448-451 (2004).

14 Maor, Y. *et al.* Clinical features of heteroresistant vancomycin-intermediate Staphylococcus aureus bacteremia versus those of methicillin-resistant S. aureus bacteremia. *The Journal of infectious diseases* **199**, 619-624 (2009).

15 Zhang, S., Sun, X., Chang, W., Dai, Y. & Ma, X. Systematic review and meta-analysis of the epidemiology of vancomycin-intermediate and heterogeneous vancomycin-intermediate Staphylococcus aureus isolates. *PloS one* **10**, e0136082 (2015).

16 Abdel-Maksoud, M. *et al.* Methicillin-Resistant Staphylococcus aureus Recovered from Healthcare- and Community-Associated Infections in Egypt. *Int J Bacteriol* **2016**, 5751785, doi:10.1155/2016/5751785 (2016).

17 AbdEl-Mongy, M., Awad, E. T. & Mosaed, F. Vancomycin Resistance Among Methicillin Resistant Staphylococcus aureus Isolates from Neonatal Sepsis Attending Intensive Care Unit in Shibin El-Kom Teaching Hospital, Egypt. *Journal of Pure and Applied Microbiology* **12**, 1093-1100 (2018).

18 Adam, H. J. *et al.* Detection and characterization of heterogeneous vancomycin-intermediate Staphylococcus aureus isolates in Canada: results from the Canadian Nosocomial Infection Surveillance Program, 1995-2006. *Antimicrob Agents Chemother* **54**, 945-949, doi:10.1128/aac.01316-09 (2010).

19 Aligholi, M. *et al.* Emergence of high-level vancomycin-resistant Staphylococcus aureus in the Imam Khomeini Hospital in Tehran. *Medical Principles and Practice* **17**, 432-434 (2008).

20 Al-Obeid, S., Haddad, Q., Cherkaoui, A., Schrenzel, J. & Francois, P. First detection of an invasive Staphylococcus aureus strain (D958) with reduced susceptibility to glycopeptides in Saudi Arabia. *Journal of clinical microbiology* **48**, 2199-2204 (2010).

21 Alzolibani, A. A. *et al.* Documentation of vancomycin-resistant Staphylococcus aureus (VRSA) among children with atopic dermatitis in the Qassim region, Saudi Arabia. *Acta Dermatovenerol Alp Pannonica Adriat* **21**, 51-53 (2012).

22 Amod, F. *et al.* Ventriculitis due to a hetero strain of vancomycin intermediate Staphylococcus aureus (hVISA): successful treatment with linezolid in combination with intraventricular vancomycin. *Journal of Infection* **50**, 252-257 (2005).

23 Andrade-Baiocchi, S., Tognim, M. C. B., Baiocchi, O. C. & Sader, H. S. Endocarditis due to glycopeptide-intermediate Staphylococcus aureus: case report and strain characterization. *Diagnostic microbiology and infectious disease* **45**, 149-152 (2003).

24 Anvari, M., Ranji, N. & Khoshmaslak, F. Antibacterial Susceptibility of Three Vancomycin-Resistant Staphylococc us aureus Strain Isolated from Nor thern Part of Iran. *J Pure Appl Microbiol* **6**, 671-675 (2012).

25 Ariza, J. & Pujol, M. Vancomycin in surgical infections due to meticillin-resistant Staphylococcus aureus with heterogeneous resistance to vancomycin. *The Lancet* **353**, 1587-1588 (1999).

26 Asadpour, L. & Ghazanfari, N. Detection of vancomycin nonsusceptible strains in clinical isolates of Staphylococcus aureus in northern Iran. *International Microbiology*, 1-7 (2019).

27 Avery, L. M., Steed, M. E., Woodruff, A. E., Hasan, M. & Rybak, M. J. Daptomycin-nonsusceptible vancomycin-intermediate Staphylococcus aureus vertebral osteomyelitis cases complicated by bacteremia treated with high-dose daptomycin and trimethoprim-sulfamethoxazole. *Antimicrobial agents and chemotherapy* **56**, 5990-5993 (2012).

28 Azimian, A. *et al.* Genetic characterization of a vancomycin-resistant Staphylococcus aureus isolate from the respiratory tract of a patient in a university hospital in northeastern Iran. *Journal of clinical microbiology* **50**, 3581-3585 (2012).

29 Backo, M., Gaenger, E., Burkart, A., Chai, Y. L. & Bayer, A. S. Treatment of experimental staphylococcal endocarditis due to a strain with reduced susceptibility in vitro to vancomycin: efficacy of ampicillin-sulbactam. *Antimicrobial agents and chemotherapy* **43**, 2565-2568 (1999).

30 Bakthavatchalam, Y. D., Ralph, R., Veeraraghavan, B., Babu, P. & Munusamy, E. Evidence from an In Vitro Study: Is Oxacillin Plus Vancomycin a Better Choice for Heteroresistant Vancomycin-Intermediate Staphylococcus aureus? *Infect Dis Ther* **8**, 51-62, doi:10.1007/s40121-018-0224-z (2019).

31 Balkhair, A., Al Muharrmi, Z., Darwish, L., Farhan, H. & Sallam, M. Treatment of vancomycin-intermediate Staphylococcus aureus (VISA) endocarditis with linezolid. *International Journal of Infectious Diseases* **14**, e227-e229 (2010).

32 Bamigboye, B. T., Olowe, O. A. & Taiwo, S. S. Phenotypic and Molecular Identification of Vancomycin Resistance in Clinical Staphylococcus Aureus Isolates in Osogbo, Nigeria. *Eur J Microbiol Immunol (Bp)* **8**, 25-30, doi:10.1556/1886.2018.00003 (2018).

33 Banerjee, T. & Anupurba, S. Colonization with vancomycin-intermediate Staphylococcus aureus strains containing the vanA resistance gene in a tertiary-care center in north India. *J Clin Microbiol* **50**, 1730-1732, doi:10.1128/jcm.06208-11 (2012).

34 Bataineh, H. A. Resistance of staphyiococcus aureus to vancomycin in Zarqa, Jordan. *Pakistan Journal of Medical Sciences* **22**, 144 (2006).

35 Baxi, S. M., Chan, D. & Jain, V. Daptomycin non-susceptible, vancomycin-intermediate Staphylococcus aureus endocarditis treated with ceftaroline and daptomycin: case report and brief review of the literature. *Infection* **43**, 751-754 (2015).

36 Bert, F. *et al.* Prevalence, molecular epidemiology, and clinical significance of heterogeneous glycopeptide-intermediate Staphylococcus aureus in liver transplant recipients. *Journal of clinical microbiology* **41**, 5147-5152 (2003).

37 Beydoun, K. & Wenzel, R. Left ventricular assist device endocarditis caused by vancomycin-intermediate Staphylococcus aureus successfully treated with ceftaroline: a review of the clinical case and overview of vancomycin resistance in Staphylococcus aureus. *Clinical Microbiology Newsletter* **35**, 171-176 (2013).

38 Bhowmick, T., Liu, C., Imp, B., Sharma, R. & Boruchoff, S. E. Ceftaroline as salvage therapy for complicated MRSA bacteremia: case series and analysis. *Infection*, 1-7 (2019).

39 Bierbaum, G., Fuchs, K., Lenz, W., Szekat, C. & Sahl, H.-G. Presence of Staphylococcus aureus with reduced susceptibility to vancomycin in Germany. *European Journal of Clinical Microbiology and Infectious Diseases* **18**, 691-696 (1999).

40 Bobin-Dubreux, S. *et al.* Clinical Isolate of Vancomycin-HeterointermediateStaphylococcus aureus Susceptible to Methicillin and In Vitro Selection of a Vancomycin-Resistant Derivative. *Antimicrobial agents and chemotherapy* **45**, 349-352 (2001).

41 Cafiso, V. *et al.* Methicillin resistance and vancomycin heteroresistance in Staphylococcus aureus in cystic fibrosis patients. *Eur J Clin Microbiol Infect Dis* **29**, 1277-1285, doi:10.1007/s10096-010-1000-5 (2010).

42 Campanile, F. *et al.* Heteroresistance to glycopeptides in Italian meticillin-resistant Staphylococcus aureus (MRSA) isolates. *Int J Antimicrob Agents* **36**, 415-419, doi:10.1016/j.ijantimicag.2010.06.044 (2010).

43 Cartolano, G. L., Cheron, M., Benabid, D., Leneveu, M. & Boisivon, A. Methicillin-resistant Staphylococcus aureus (MRSA) with reduced susceptibility to glycopeptides (GISA) in 63 French general hospitals. *Clin Microbiol Infect* **10**, 448-451, doi:10.1111/j.1469-0691.2004.00830.x (2004).

44 Casapao, A. M. *et al.* Evaluation of vancomycin population susceptibility analysis profile as a predictor of outcomes for patients with infective endocarditis due to methicillin-resistant Staphylococcus aureus. *Antimicrob Agents Chemother* **58**, 4636-4641, doi:10.1128/aac.02820-13 (2014).

45 Cha, H. Y., Kim, H. O., Jin, J. S. & Lee, J. C. Emergence of vancomycin-intermediate Staphylococcus aureus from predominant methicillin-resistant S. aureus clones in a Korean hospital. *The Journal of Microbiology* **48**, 533-535 (2010).

46 Chaiwongkarjohn, S. *et al.* The First Case of Vancomycin-Intermediate Staphylococcus aureus in Hawai'i. *Hawaii Medical Journal* **68** (2009).

47 Chaiwongkarjohn, S. *et al.* A Report on The First Case of Vancomycin-Intermediate Staphylococcus aureus (VISA) in Hawai ‘i. *Hawaii medical journal* **70**, 233 (2011).

48 Chang, S. *et al.* Infection with vancomycin-resistant Staphylococcus aureus containing the vanA resistance gene. *New England Journal of Medicine* **348**, 1342-1347 (2003).

49 Chaudhari, C. *et al.* Heterogeneous vancomycin-intermediate among methicillin resistant Staphylococcus aureus. *medical journal armed forces india* **71**, 15-18 (2015).

50 Chaudhary, M. & Payasi, A. Prevalence of heterogeneous glycopeptide intermediate resistance in Methicillin-Resistant Staphylococcus aureus. *American Journal of Infectious Diseases* **9**, 63 (2013).

51 Chaudhary, M. & Payasi, A. Vancoplus Kinetic Study in Vancomycin Resistant Staphylococcus aureus. *International Journal of Pharmaceutical Sciences Review and Research* **31**, 135-142 (2015).

52 Chen, C.-J., Lin, M.-H., Shu, J.-C. & Lu, J.-J. Reduced susceptibility to vancomycin in isogenic Staphylococcus aureus strains of sequence type 59: tracking evolution and identifying mutations by whole-genome sequencing. *Journal of Antimicrobial Chemotherapy* **69**, 349-354 (2013).

53 Chua, T. *et al.* Molecular epidemiology of methicillin-resistant Staphylococcus aureus bloodstream isolates in urban Detroit. *J Clin Microbiol* **46**, 2345-2352, doi:10.1128/jcm.00154-08 (2008).

54 Chung, D. R. *et al.* Genotype-specific prevalence of heterogeneous vancomycin-intermediate Staphylococcus aureus in Asian countries. *Int J Antimicrob Agents* **46**, 338-341, doi:10.1016/j.ijantimicag.2015.03.009 (2015).

55 Chung, G. *et al.* Nationwide surveillance study of vancomycin intermediate Staphylococcus aureus strains in Korean hospitals from 2001 to 2006. *J Microbiol Biotechnol* **20**, 637-642 (2010).

56 Chung, G. *et al.* Nationwide surveillance Study of vancomycin-intermediate Staphylococcus aureus strains in Korean Hospitals from 2001 to 2006. (2010).

57 Claeys, K. C. *et al.* Pneumonia Caused by Methicillin-Resistant Staphylococcus aureus: Does Vancomycin Heteroresistance Matter? *Antimicrob Agents Chemother* **60**, 1708-1716, doi:10.1128/aac.02388-15 (2016).

58 da Costa, T. M. *et al.* Clinical and Microbiological Characteristics of Heteroresistant and Vancomycin-Intermediate Staphylococcus aureus from Bloodstream Infections in a Brazilian Teaching Hospital. *PLoS One* **11**, e0160506, doi:10.1371/journal.pone.0160506 (2016).

59 Dedania, V. S., Hale, B. P. & Bhatnagar, P. Endogenous endophthalmitis due to clinically vancomycin-resistant Staphylococcus aureus. *Retinal Cases and Brief Reports* **9**, 59-60 (2015).

60 Denis, O., Deplano, A., De Ryck, R., Nonhoff, C. & Struelens, M. J. Emergence and spread of gentamicin-susceptible strains of methicillin-resistant Staphylococcus aureus in Belgian hospitals. *Microb Drug Resist* **9**, 61-71, doi:10.1089/107662903764736355 (2003).

61 Denis, O. *et al.* Emergence of vancomycin-intermediate Staphylococcus aureus in a Belgian hospital: microbiological and clinical features. *J Antimicrob Chemother* **50**, 383-391, doi:10.1093/jac/dkf142 (2002).

62 Dezfulian, A. *et al.* Identification and characterization of a high vancomycin-resistant Staphylococcus aureus harboring VanA gene cluster isolated from diabetic foot ulcer. *Iranian journal of basic medical sciences* **15**, 803 (2012).

63 Di Gregorio, S. *et al.* Clinical, microbiological, and genetic characteristics of heteroresistant vancomycin-intermediate Staphylococcus aureus bacteremia in a teaching hospital. *Microb Drug Resist* **21**, 25-34, doi:10.1089/mdr.2014.0190 (2015).

64 Dubey, D. *et al.* Surveillance of infection status of drug resistant Staphylococcus aureus in an Indian teaching hospital. *Asian Pacific journal of tropical disease* **3**, 133-142 (2013).

65 El Ayoubi, M.-D., Hamze, M., Mallat, H., Achkar, M. & Dabboussi, F. Glycopeptide intermediate Staphylococcus aureus and prevalence of the luk-PV gene in clinical isolates, in Northern Lebanon. *Médecine et Maladies Infectieuses* **44**, 223-228 (2014).

66 El-Aziz, N. K. A., El-Hamid, M. I. A., Bendary, M. M., El-Azazy, A. A. & Ammar, A. M. Existence of vancomycin resistance among methicillin resistant S. aureus recovered from animal and human sources in Egypt. *Veterinary Medicine In-between Health & Economy (VMHE)–16-19 October 2018* **55** (2018).

67 ElSayed, N., Ashour, M. & Amine, A. E. K. Vancomycin resistance among Staphylococcus aureus isolates in a rural setting, Egypt. *Germs* **8**, 134 (2018).

68 Fasihi, Y., Kiaei, S. & Kalantar-Neyestanaki, D. Characterization of SCCmec and spa types of methicillin-resistant Staphylococcus aureus isolates from health-care and community-acquired infections in Kerman, Iran. *J Epidemiol Glob Health* **7**, 263-267, doi:10.1016/j.jegh.2017.08.004 (2017).

69 Fasihi, Y., Saffari, F., Mansouri, S. & Kalantar-Neyestanaki, D. The emergence of vancomycin-resistant Staphylococcus aureus in an intensive care unit in Kerman, Iran. *Wien Med Wochenschr* **168**, 85-88, doi:10.1007/s10354-017-0562-6 (2018).

70 Finks, J. *et al.* Vancomycin-resistant Staphylococcus aureus, Michigan, USA, 2007. *Emerging infectious diseases* **15**, 943 (2009).

71 Fitzgibbon, M. M., Rossney, A. S. & O'Connell, B. Investigation of reduced susceptibility to glycopeptides among methicillin-resistant Staphylococcus aureus isolates from patients in Ireland and evaluation of agar screening methods for detection of heterogeneously glycopeptide-intermediate S. aureus. *J Clin Microbiol* **45**, 3263-3269, doi:10.1128/jcm.00836-07 (2007).

72 Fong, R. K., Low, J., Koh, T. H. & Kurup, A. Clinical features and treatment outcomes of vancomycin-intermediate Staphylococcus aureus (VISA) and heteroresistant vancomycin-intermediate Staphylococcus aureus (hVISA) in a tertiary care institution in Singapore. *Eur J Clin Microbiol Infect Dis* **28**, 983-987, doi:10.1007/s10096-009-0741-5 (2009).

73 Gagliotti, C. *et al.* Staphylococcus aureus in a northern Italian region: phenotypic and molecular characterization. *Scandinavian journal of infectious diseases* **44**, 24-28 (2012).

74 Gallon, O. *et al.* Antimicrobial susceptibility profiles of Staphylococcus aureus isolated in 2007 from French patients with bloodstream infections: goodbye hVISA, welcome Geraldine? *Journal of Antimicrobial Chemotherapy* **65**, 1297-1299 (2010).

75 Gardete, S., Aires-De-Sousa, M., Faustino, A., Ludovice, A. & de Lencastre, H. Identification of the first vancomycin intermediate-resistant Staphylococcus aureus (VISA) isolate from a hospital in Portugal. *Microbial Drug Resistance* **14**, 1-6 (2008).

76 Garnier, F. *et al.* A 1 year surveillance study of glycopeptide-intermediate Staphylococcus aureus strains in a French hospital. *J Antimicrob Chemother* **57**, 146-149, doi:10.1093/jac/dki413 (2006).

77 Gecgel, A. S. K. Vancomycin Minimum Inhibitory Concentration (MIC) Creep and Its Effect on Mortality in Adult Cardiac Patients Who Developed Sepsis Caused by Staphylococcus Aureus and Coagulase-Negative Staphylococcus. (2017).

78 Ghahremani, M., Jazani, N. H. & Sharifi, Y. Emergence of vancomycin-intermediate and -resistant Staphylococcus aureus among methicillin-resistant S. aureus isolated from clinical specimens in the northwest of Iran. *J Glob Antimicrob Resist* **14**, 4-9, doi:10.1016/j.jgar.2018.01.017 (2018).

79 Goud, R. *et al.* Community prevalence of methicillin and vancomycin resistant Staphylococcus aureus in and around Bangalore, southern India. *Rev Soc Bras Med Trop* **44**, 309-312, doi:10.1590/s0037-86822011005000035 (2011).

80 Gowrishankar, S., Thenmozhi, R., Balaji, K. & Pandian, S. K. Emergence of methicillin-resistant, vancomycin-intermediate Staphylococcus aureus among patients associated with group A Streptococcal pharyngitis infection in southern India. *Infect Genet Evol* **14**, 383-389, doi:10.1016/j.meegid.2013.01.002 (2013).

81 Hafer, C., Lin, Y., Kornblum, J., Lowy, F. D. & Uhlemann, A. C. Contribution of selected gene mutations to resistance in clinical isolates of vancomycin-intermediate Staphylococcus aureus. *Antimicrob Agents Chemother* **56**, 5845-5851, doi:10.1128/aac.01139-12 (2012).

82 Hageman, J. C. *et al.* Occurrence of a USA300 vancomycin-intermediate Staphylococcus aureus. *Diagnostic microbiology and infectious disease* **62**, 440-442 (2008).

83 Hageman, J. C. *et al.* Vancomycin-intermediate Staphylococcus aureus in a home health-care patient. *Emerging infectious diseases* **7**, 1023 (2001).

84 Hakim, S., Arshed, S., Iqbal, M. & Javaid, S. Vancomycin sensitivity of Staphylococcus aureus isolates from hospital patients in Karachi, Pakistan. *Libyan Journal of medicine* **2**, 176-179 (2007).

85 Hala Ibrahim Al Al, D., Walid, D. S., Muna, A.-k. & Saleh, M. A. A.-l. Asymptomatic colonization of Staphylococcus aureus with intermediate resistance to vancomycin harboring vanB resistance gene. *Asian Journal of Pharmaceutical and Clinical Research* **10**, doi:10.22159/ajpcr.2017.v10i5.17285 (2017).

86 Hanaki, H. *et al.* Antibiotic susceptibility survey of blood-borne MRSA isolates in Japan from 2008 through 2011. *J Infect Chemother* **20**, 527-534, doi:10.1016/j.jiac.2014.06.012 (2014).

87 Hanaki, H. *et al.* Occurrence of vancomycin-intermediate-resistant Staphylococcus aureus in Japan. *J Infect Chemother* **13**, 118-121, doi:10.1007/s10156-006-0498-z (2007).

88 Hasan, R., Acharjee, M. & Noor, R. Prevalence of vancomycin resistant Staphylococcus aureus (VRSA) in methicillin resistant S. aureus (MRSA) strains isolated from burn wound infections. *Ci Ji Yi Xue Za Zhi* **28**, 49-53, doi:10.1016/j.tcmj.2016.03.002 (2016).

89 Havaei, S. A. *et al.* Genetic characterization of methicillin resistant and sensitive, vancomycin intermediate Staphylococcus aureus strains isolated from different Iranian Hospitals. *ISRN microbiology* **2012** (2012).

90 Ho, C. M. *et al.* Prevalence and accessory gene regulator (agr) analysis of vancomycin-intermediate Staphylococcus aureus among methicillin-resistant isolates in Taiwan--SMART program, 2003. *Eur J Clin Microbiol Infect Dis* **29**, 383-389, doi:10.1007/s10096-009-0868-4 (2010).

91 Hong, K. H., Park, J. S. & Kim, E.-C. Two cases of vancomycin-intermediate Staphylococcus aureus isolated from joint tissue or wound. *The Korean journal of laboratory medicine* **28**, 444-448 (2008).

92 Horne, K. C. *et al.* Prospective comparison of the clinical impacts of heterogeneous vancomycin-intermediate methicillin-resistant Staphylococcus aureus (MRSA) and vancomycin-susceptible MRSA. *Antimicrobial agents and chemotherapy* **53**, 3447-3452 (2009).

93 Hsueh, P. R., Lee, S. Y., Perng, C. L., Chang, T. Y. & Lu, J. J. Clonal dissemination of meticillin-resistant and vancomycin-intermediate Staphylococcus aureus in a Taiwanese hospital. *Int J Antimicrob Agents* **36**, 307-312, doi:10.1016/j.ijantimicag.2010.06.035 (2010).

94 Hu, J. *et al.* Reduced vancomycin susceptibility found in methicillin-resistant and methicillin-sensitive Staphylococcus aureus clinical isolates in Northeast China. *PLoS One* **8**, e73300, doi:10.1371/journal.pone.0073300 (2013).

95 Huang, S. H. *et al.* Prevalence of vancomycin-intermediate Staphylococcus aureus (VISA) and heterogeneous VISA among methicillin-resistant S. aureus with high vancomycin minimal inhibitory concentrations in Taiwan: A multicenter surveillance study, 2012-2013. *Journal of microbiology, immunology, and infection = Wei mian yu gan ran za zhi* **49**, 701-707, doi:10.1016/j.jmii.2015.07.003 (2016).

96 Hubert, S. K. *et al.* Glycopeptide-intermediate Staphylococcus aureus: evaluation of a novel screening method and results of a survey of selected U.S. hospitals. *J Clin Microbiol* **37**, 3590-3593 (1999).

97 Islam, T. A. B. & Shamsuzzaman, S. Prevalence and antimicrobial susceptibility pattern of methicillin-resistant, vancomycin-resistant, and Panton-Valentine leukocidin positive Staphylococcus aureus in a tertiary care hospital Dhaka, Bangladesh. *Tzu Chi Medical Journal* **27**, 10-14 (2015).

98 Jahanshahi, A., Zeighami, H. & Haghi, F. Molecular Characterization of Methicillin and Vancomycin Resistant Staphylococcus aureus Strains Isolated from Hospitalized Patients. *Microb Drug Resist*, doi:10.1089/mdr.2018.0069 (2018).

99 Julian, K. *et al.* Characterization of a daptomycin-nonsusceptible vancomycin-intermediate Staphylococcus aureus strain in a patient with endocarditis. *Antimicrobial agents and chemotherapy* **51**, 3445-3448 (2007).

100 Kang, Y. R. *et al.* Decreasing prevalence of heterogeneous vancomycin-intermediate Staphylococcus aureus among blood isolates in Korean hospitals. *Diagn Microbiol Infect Dis* **86**, 464-466, doi:10.1016/j.diagmicrobio.2016.09.015 (2016).

101 Khatib, R. *et al.* Relevance of vancomycin-intermediate susceptibility and heteroresistance in methicillin-resistant Staphylococcus aureus bacteraemia. *J Antimicrob Chemother* **66**, 1594-1599, doi:10.1093/jac/dkr169 (2011).

102 Khatib, R. *et al.* Decreasing prevalence of isolates with vancomycin heteroresistance and vancomycin minimum inhibitory concentrations >/=2 mg/L in methicillin-resistant Staphylococcus aureus over 11 years: potential impact of vancomycin treatment guidelines. *Diagn Microbiol Infect Dis* **82**, 245-248, doi:10.1016/j.diagmicrobio.2015.03.014 (2015).

103 Khosrovaneh, A. *et al.* Frequency of reduced vancomycin susceptibility and heterogeneous subpopulation in persistent or recurrent methicillin-resistant Staphylococcus aureus bacteremia. *Clinical infectious diseases* **38**, 1328-1330 (2004).

104 Kim, E. S. *et al.* Clinical and Molecular Characterization of Invasive Heteroresistant Vancomycin-Intermediate Staphylococcus aureus Infections in Korean Hospitals. *J Clin Microbiol* **54**, 760-763, doi:10.1128/jcm.02595-15 (2016).

105 Kim, M.-N., Pai, C. H., Woo, J. H., Ryu, J. S. & Hiramatsu, K. Vancomycin-intermediate Staphylococcus aureus in Korea. *Journal of clinical microbiology* **38**, 3879-3881 (2000).

106 Kim, T. *et al.* Phenotypic changes of methicillin-resistant Staphylococcus aureus during vancomycin therapy for persistent bacteraemia and related clinical outcome. *Eur J Clin Microbiol Infect Dis* **36**, 1473-1481, doi:10.1007/s10096-017-2956-1 (2017).

107 Kino, H. *et al.* Central nervous system infection caused by vancomycin-intermediate Staphylococcus aureus (SCCmec type IV, ST8). *Journal of Infection and Chemotherapy* **20**, 643-646 (2014).

108 Kirby, A. *et al.* Staphylococcus aureus with reduced glycopeptide susceptibility in Liverpool, UK. *J Antimicrob Chemother* **65**, 721-724, doi:10.1093/jac/dkq009 (2010).

109 Koh, Y. R., Kim, K. H., Chang, C. L. & Yi, J. Prevalence and Clinical Impact of Heterogeneous Vancomycin-Intermediate Staphylococcus aureus Isolated From Hospitalized Patients. *Ann Lab Med* **36**, 235-243, doi:10.3343/alm.2016.36.3.235 (2016).

110 Kosowska-Shick, K. *et al.* Incidence and characteristics of vancomycin nonsusceptible strains of methicillin-resistant Staphylococcus aureus at Hershey Medical Center. *Antimicrobial agents and chemotherapy* **52**, 4510-4513 (2008).

111 Krzyszton-Russjan, J., Gniadkowski, M., Polowniak-Pracka, H., Hagmajer, E. & Hryniewicz, W. The first Staphylococcus aureus isolates with reduced susceptibility to vancomycin in Poland. *J Antimicrob Chemother* **50**, 1065-1069, doi:10.1093/jac/dkf252 (2002).

112 Kumar, M. Multidrug-Resistant Staphylococcus aureus, India, 2013-2015. *Emerg Infect Dis* **22**, 1666-1667, doi:10.3201/eid2209.160044 (2016).

113 Lee, H.-W. *et al.* Detection of MecA gene in clinical isolates of Staphylococcus aureus by multiplex-PCR, and antimicrobial susceptibility of MRSA. *Journal of microbiology and biotechnology* **13**, 354-359 (2003).

114 Liaqat, F. *et al.* Isolation identification and control of vancomycin resistant Staphylococcus aureus. *Pak J Pharm Sci* **28**, 997-1004 (2015).

115 Limbago, B. M. *et al.* Report of the 13th vancomycin-resistant Staphylococcus aureus isolate from the United States. *Journal of clinical microbiology* **52**, 998-1002 (2014).

116 Lin, S. Y. *et al.* Molecular epidemiology and clinical characteristics of hetero-resistant vancomycin intermediate Staphylococcus aureus bacteremia in a Taiwan Medical Center. *Journal of microbiology, immunology, and infection = Wei mian yu gan ran za zhi* **45**, 435-441, doi:10.1016/j.jmii.2012.05.004 (2012).

117 Liu, C. *et al.* Molecular characteristics and virulence factors in methicillin-susceptible, resistant, and heterogeneous vancomycin-intermediate Staphylococcus aureus from central-southern China. *Journal of microbiology, immunology, and infection = Wei mian yu gan ran za zhi* **48**, 490-496, doi:10.1016/j.jmii.2014.03.003 (2015).

118 Lulitanond, A. *et al.* The first vancomycin-intermediate Staphylococcus aureus strains isolated from patients in Thailand. *Journal of clinical microbiology* **47**, 2311-2316 (2009).

119 Mabed, M. & Marouf, S. Vancomycin-resistant Staphylococcus aureus in a bone marrow transplantation unit. *Annals of hematology* **84**, 133-135 (2005).

120 Maor, Y. *et al.* Prevalence and characteristics of heteroresistant vancomycin-intermediate Staphylococcus aureus bacteremia in a tertiary care center. *J Clin Microbiol* **45**, 1511-1514, doi:10.1128/jcm.01262-06 (2007).

121 Marchese, A., Balistreri, G., Tonoli, E., Debbia, E. & Schito, G. Heterogeneous Vancomycin Resistance in Methicillin-ResistantStaphylococcus aureus Strains Isolated in a Large Italian Hospital. *Journal of clinical microbiology* **38**, 866-869 (2000).

122 Martirosov, D. M. *et al.* Relationship between day 1 and day 2 Vancomycin area under the curve values and emergence of heterogeneous Vancomycin-intermediate Staphylococcus aureus (hVISA) by Etest(R) macromethod among patients with MRSA bloodstream infections: a pilot study. *BMC Infect Dis* **17**, 534, doi:10.1186/s12879-017-2609-0 (2017).

123 Melo, G. B. *et al.* Analysis of the genetic diversity of vancomycin-resistant Staphylococcus aureus. *Brazilian Journal of Microbiology* **36**, 126-130 (2005).

124 Melo-Cristino, J., Resina, C., Manuel, V., Lito, L. & Ramirez, M. First case of infection with vancomycin-resistant Staphylococcus aureus in Europe. *The Lancet* **382**, 205 (2013).

125 Mendem, S. K., Alasthimannahalli Gangadhara, T., Shivannavar, C. T. & Gaddad, S. M. Antibiotic resistance patterns of Staphylococcus aureus: A multi center study from India. *Microb Pathog* **98**, 167-170, doi:10.1016/j.micpath.2016.07.010 (2016).

126 Mirani, Z. A. & Jamil, N. Characterization of a vancomycin intermediate-resistant Staphylococcus aureus isolated from a hospital. *Journal of the College of Physicians and Surgeons Pakistan* **20**, 558-559 (2010).

127 Mirza, H. C., Sancak, B. & Gur, D. The Prevalence of Vancomycin-Intermediate Staphylococcus aureus and Heterogeneous VISA Among Methicillin-Resistant Strains Isolated from Pediatric Population in a Turkish University Hospital. *Microb Drug Resist* **21**, 537-544, doi:10.1089/mdr.2015.0048 (2015).

128 Mlynarczyk, A., Mlynarczyk, G. & Luczak, M. [Searching for Staphylococcus aureus strains with reduced susceptibility to glycopeptides among clinical isolates obtained during the year of 2002]. *Med Dosw Mikrobiol* **55**, 209-217 (2003).

129 Monaco, M., Sanchini, A., Grundmann, H. & Pantosti, A. Vancomycin-heteroresistant phenotype in invasive methicillin-resistant Staphylococcus aureus isolates belonging to spa type 041. *Eur J Clin Microbiol Infect Dis* **29**, 771-777, doi:10.1007/s10096-010-0922-2 (2010).

130 Muneeri, S. S., Mobaiyen, H. & Mirzaie, H. Study on Vancomycin-resistant staphylococcus aureus and identification of VanA gene in these strains isolated from Tabriz Shuhada Hospital using e-test and PCR methods. *Life Sci J* **10**, 748-752 (2013).

131 Musta, A. C. *et al.* Vancomycin MIC plus heteroresistance and outcome of methicillin-resistant Staphylococcus aureus bacteremia: trends over 11 years. *J Clin Microbiol* **47**, 1640-1644, doi:10.1128/jcm.02135-08 (2009).

132 Naimi, T. S. *et al.* Vancomycin-intermediate Staphylococcus aureus with phenotypic susceptibility to methicillin in a patient with recurrent bacteremia. *Clinical infectious diseases* **36**, 1609-1612 (2003).

133 Najar-Peerayeh, S., Mirzaee, M. & Behmanesh, M. Molecular characterization of vancomycin-intermediate Staphylococcus aureus isolates from Tehran. *Asian Pacific Journal of Tropical Disease* **6**, 726-731 (2016).

134 Neetu, T. J. P. & Murugan, S. Genotyping of methicillin resistant Staphylococcus aureus from tertiary care hospitals in Coimbatore, South India. *Journal of global infectious diseases* **8**, 68 (2016).

135 Neoh, H.-m. *et al.* Impact of reduced vancomycin susceptibility on the therapeutic outcome of MRSA bloodstream infections. *Annals of Clinical Microbiology and Antimicrobials* **6**, 13 (2007).

136 Nonhoff, C., Denis, O. & Struelens, M. Low prevalence of methicillin-resistant Staphylococcus aureus with reduced susceptibility to glycopeptides in Belgian hospitals. *Clinical microbiology and infection* **11**, 214-220 (2005).

137 Norazah, A., Law, N. L., Kamel, A. G. & Salbiah, N. The presence of heterogeneous vancomycin-lntermediate Staphylococcus aureus (heteroVISA) in a major Malaysian hospital. *Med J Malaysia* **67**, 269-273 (2012).

138 Oguz, V. A., Kose, H., Yapar, N., Karatosun, V. & Gulay, Z. Heteroresistant vancomycin intermediate S. aureus (h-VISA) isolated from a patient with orthopedic implant infection treated with glycopeptides: A case report. *Journal of Experimental and Clinical Medicine* **34**, 149-154 (2017).

139 Okada, N. *et al.* A case report of postoperative VRSA enteritis: Effective management of rifampicin for vancomycin resistant Staphylococcus aureus enteritis after esophagectomy and colon reconstruction. *International journal of surgery case reports* **52**, 75-78 (2018).

140 Oksuz, L. *et al.* The high diversity of MRSA clones detected in a university hospital in Istanbul. *International journal of medical sciences* **10**, 1740 (2013).

141 Oliveira, G. A. *et al.* Isolation in Brazil of nosocomial Staphylococcus aureus with reduced susceptibility to vancomycin. *Infection Control & Hospital Epidemiology* **22**, 443-448 (2001).

142 Olufunmiso, O., Tolulope, I. & Roger, C. Multidrug and vancomycin resistance among clinical isolates of Staphylococcus aureus from different teaching hospitals in Nigeria. *African health sciences* **17**, 797-807 (2017).

143 Othman, H. B., Halim, R. M. A., Gomaa, F. A. M. & Amer, M. Z. Vancomycin MIC Distribution among Methicillin-Resistant Staphylococcus Aureus. Is Reduced Vancomycin Susceptibility Related To MIC Creep? *Open Access Maced J Med Sci* **7**, 12-18, doi:10.3889/oamjms.2019.009 (2019).

144 Ouko, T. T. *et al.* Oxacillin resistant Staphylococcus aureus among HIV infected and non-infected Kenyan patients. *East Afr Med J* **87**, 179-186 (2010).

145 Panesso, D. *et al.* Methicillin-susceptible, vancomycin-resistant Staphylococcus aureus, Brazil. *Emerging infectious diseases* **21**, 1844 (2015).

146 Parer, S. *et al.* An outbreak of heterogeneous glycopeptide-intermediate Staphylococcus aureus related to a device source in an intensive care unit. *Infect Control Hosp Epidemiol* **33**, 167-174, doi:10.1086/663703 (2012).

147 Park, K. H. *et al.* Comparison of the clinical features, bacterial genotypes and outcomes of patients with bacteraemia due to heteroresistant vancomycin-intermediate Staphylococcus aureus and vancomycin-susceptible S. aureus. *J Antimicrob Chemother* **67**, 1843-1849, doi:10.1093/jac/dks131 (2012).

148 Park, M. J. *et al.* Accessory Gene Regulator Polymorphism and Vancomycin Minimum Inhibitory Concentration in Methicillin-Resistant Staphylococcus aureus. *Ann Lab Med* **35**, 399-403, doi:10.3343/alm.2015.35.4.399 (2015).

149 Phongsamart, W. *et al.* The first pediatric case of Staphylococcus aureus with heterogenous resistant to vancomycin endocarditis in Thailand. *J Med Assoc Thai* **88**, S264-268 (2005).

150 Pierard, D., Vandenbussche, H., Verschraegen, I. & Lauwers, S. [Screening for Staphylococcus aureus with a reduced susceptibility to vancomycin in: a Belgian hospital]. *Pathol Biol (Paris)* **52**, 486-488, doi:10.1016/j.patbio.2004.07.016 (2004).

151 Pitz, A. M. *et al.* Vancomycin susceptibility trends and prevalence of heterogeneous vancomycin-intermediate Staphylococcus aureus in clinical methicillin-resistant S. aureus isolates. *J Clin Microbiol* **49**, 269-274, doi:10.1128/jcm.00914-10 (2011).

152 Ramli, S. R., Neoh, H. M., Aziz, M. N. & Hussin, S. Screening and detection of heterogenous vancomycin intermediate Staphylococcus aureus in Hospital Kuala Lumpur Malaysia, using the glycopeptide resistance detection Etest and population analysis profiling. *Infect Dis Rep* **4**, e20, doi:10.4081/idr.2012.e20 (2012).

153 Rebiahi, S., Abdelouahid, D., Rahmoun, M., Abdelali, S. & Azzaoui, H. Emergence of vancomycin-resistant Staphylococcus aureus identified in the Tlemcen university hospital (North-West Algeria). *Médecine et maladies infectieuses* **41**, 646-651 (2011).

154 Reverdy, M. *et al.* Incidence of Staphylococcus aureus with reduced susceptibility to glycopeptides in two French hospitals. *Clinical microbiology and infection* **7**, 267-272 (2001).

155 Richter, S. S. *et al.* Activities of vancomycin, ceftaroline, and mupirocin against Staphylococcus aureus isolates collected in a 2011 national surveillance study in the United States. *Antimicrobial agents and chemotherapy* **58**, 740-745 (2014).

156 Richter, S. S. *et al.* Detection of Staphylococcus aureus isolates with heterogeneous intermediate-level resistance to vancomycin in the United States. *J Clin Microbiol* **49**, 4203-4207, doi:10.1128/jcm.01152-11 (2011).

157 Riederer, K. *et al.* Detection of intermediately vancomycin-susceptible and heterogeneous Staphylococcus aureus isolates: comparison of Etest and Agar screening methods. *J Clin Microbiol* **49**, 2147-2150, doi:10.1128/jcm.01435-10 (2011).

158 Robert, J., Bismuth, R. & Jarlier, V. Decreased susceptibility to glycopeptides in methicillin-resistant Staphylococcus aureus: a 20 year study in a large French teaching hospital, 1983-2002. *J Antimicrob Chemother* **57**, 506-510, doi:10.1093/jac/dki486 (2006).

159 Rossi, F. *et al.* Transferable vancomycin resistance in a community-associated MRSA lineage. *New England Journal of Medicine* **370**, 1524-1531 (2014).

160 Rybak, M. J. *et al.* Characterization of vancomycin-heteroresistant Staphylococcus aureus from the metropolitan area of Detroit, Michigan, over a 22-year period (1986 to 2007). *J Clin Microbiol* **46**, 2950-2954, doi:10.1128/jcm.00582-08 (2008).

161 Sader, H. S., Jones, R. N., Rossi, K. L. & Rybak, M. J. Occurrence of vancomycin-tolerant and heterogeneous vancomycin-intermediate strains (hVISA) among Staphylococcus aureus causing bloodstream infections in nine USA hospitals. *J Antimicrob Chemother* **64**, 1024-1028, doi:10.1093/jac/dkp319 (2009).

162 Sambandam, S. N., Rohinikumar, G. J., Gul, A. & Mounasamy, V. Intramuscular Injection Abscess Due to VRSA: A New Health Care Challenge. *Archives of Bone and Joint Surgery* **4**, 277 (2016).

163 Sancak, B., Ercis, S., Menemenlioglu, D., Colakoglu, S. & Hascelik, G. Methicillin-resistant Staphylococcus aureus heterogeneously resistant to vancomycin in a Turkish university hospital. *J Antimicrob Chemother* **56**, 519-523, doi:10.1093/jac/dki272 (2005).

164 Sancak, B. *et al.* Vancomycin and daptomycin minimum inhibitory concentration distribution and occurrence of heteroresistance among methicillin-resistant Staphylococcus aureus blood isolates in Turkey. *BMC Infect Dis* **13**, 583, doi:10.1186/1471-2334-13-583 (2013).

165 Shekarabi, M., Hajikhani, B., Chirani, A. S., Fazeli, M. & Goudarzi, M. Molecular characterization of vancomycin-resistant Staphylococcus aureus strains isolated from clinical samples: A three year study in Tehran, Iran. *PloS one* **12**, e0183607 (2017).

166 Silveira, A. C. *et al.* Is prediffusion test an alternative to improve accuracy in screening hVISA strains and to detect susceptibility to glycopeptides/lipopeptides? *Diagn Microbiol Infect Dis* **79**, 401-404, doi:10.1016/j.diagmicrobio.2014.04.008 (2014).

167 Singh, A. *et al.* Increasing Trend of Heterogeneous Vancomycin Intermediate Staphylococcus aureus in a Tertiary Care Center of Northern India. *Microb Drug Resist* **21**, 545-550, doi:10.1089/mdr.2015.0004 (2015).

168 Sivakumar, B., Vijaysegaran, P., Chaudhuri, A., Crawford, S. & Ottley, M. Daptomycin resistance in prosthetic joint infections. *Orthopedics* **35**, e603-e606 (2012).

169 Sng, L.-H. *et al.* Heterogeneous vancomycin-resistant Staphylococcus aureus (hetero-VISA) in Singapore. *International journal of antimicrobial agents* **25**, 177-179 (2005).

170 Sola, C. *et al.* Heterogeneous vancomycin-intermediate susceptibility in a community-associated methicillin-resistant Staphylococcus aureus epidemic clone, in a case of Infective Endocarditis in Argentina. *Annals of clinical microbiology and antimicrobials* **10**, 15 (2011).

171 Song, J. H. *et al.* Emergence in Asian countries of Staphylococcus aureus with reduced susceptibility to vancomycin. *Antimicrob Agents Chemother* **48**, 4926-4928, doi:10.1128/aac.48.12.4926-4928.2004 (2004).

172 Stroh, E. M. Quinupristin/dalfopristin in vancomycin-resistant Staphylococcus aureus endophthalmitis. *Archives of Ophthalmology* **130**, 1323-1324 (2012).

173 Sumon, Z. E. *et al.* Successful cure of daptomycin-non-susceptible, vancomycin-intermediate Staphylococcus aureus prosthetic aortic valve endocarditis directed by synergistic in vitro time-kill study. *Infectious Diseases* **51**, 287-292 (2019).

174 Sun, W. *et al.* Prevalence and characterization of heterogeneous vancomycin-intermediate Staphylococcus aureus isolates from 14 cities in China. *Antimicrob Agents Chemother* **53**, 3642-3649, doi:10.1128/aac.00206-09 (2009).

175 Swartz, T. *et al.* Heart transplantation in a patient with heteroresistant vancomycin‐intermediate S taphylococcus aureus ventricular assist device mediastinitis and bacteremia. *Transplant Infectious Disease* **15**, E177-E181 (2013).

176 Taha, A., Badr, M., El-Morsy, F. & Hammad, E. Report of β-lactam antibiotic–induced vancomycin-resistant Staphylococcus aureus from a university hospital in Egypt. *New microbes and new infections* **29**, 100507 (2019).

177 Takata, T. *et al.* Presence of both heterogeneous vancomycin-intermediate resistance and β-lactam antibiotic-induced vancomycin resistance phenotypes is associated with the outcome in methicillin-resistant Staphylococcus aureus bloodstream infection. *Scandinavian journal of infectious diseases* **45**, 203-212 (2013).

178 Tallent, S. M. *et al.* Vancomycin susceptibility of oxacillin-resistant Staphylococcus aureus isolates causing nosocomial bloodstream infections. *J Clin Microbiol* **40**, 2249-2250, doi:10.1128/jcm.40.6.2249-2250.2002 (2002).

179 Tascini, C. *et al.* Case report of a successful treatment of methicillin-resistant Staphylococcus aureus (MRSA) bacteremia and MRSA/vancomycin-resistant Enterococcus faecium cholecystitis by daptomycin. *Antimicrobial agents and chemotherapy* **55**, 2458-2459 (2011).

180 Thati, V., Shivannavar, C. T. & Gaddad, S. M. Vancomycin resistance among methicillin resistant Staphylococcus aureus isolates from intensive care units of tertiary care hospitals in Hyderabad. *The Indian journal of medical research* **134**, 704 (2011).

181 Thirat, S. Methicillin-resistant Staphylococcus aureus with reduced susceptibility to vancomycin in Sanprasitthiprasong Hospital. *J Med Assoc Thai* **97**, S1 (2014).

182 Tiwari, H. K. & Sen, M. R. Emergence of vancomycin resistant Staphylococcus aureus (VRSA) from a tertiary care hospital from northern part of India. *BMC Infectious diseases* **6**, 156 (2006).

183 Tóth, Á. *et al.* First report of heterogeneously vancomycin-intermediate Staphylococcus aureus (hVISA) causing fatal infection in Hungary. *Journal of Chemotherapy* **20**, 655-656 (2008).

184 Trakulsomboon, S. *et al.* First Report of Methicillin-ResistantStaphylococcus aureus with Reduced Susceptibility to Vancomycin in Thailand. *Journal of clinical microbiology* **39**, 591-595 (2001).

185 Tsakris, A., Papadimitriou, E., Douboyas, J., Stylianopoulou, F. & Manolis, E. Emergence of vancomycin-intermediate Staphylococcus aureus and S. sciuri, Greece. *Emerging Infectious Diseases* **8**, 536 (2002).

186 Ullah, A. *et al.* High frequency of methicillin-resistant Staphylococcus aureus in Peshawar Region of Pakistan. *Springerplus* **5**, 600, doi:10.1186/s40064-016-2277-3 (2016).

187 van Hal, S. J., Jones, M., Gosbell, I. B. & Paterson, D. L. Vancomycin heteroresistance is associated with reduced mortality in ST239 methicillin-resistant Staphylococcus aureus blood stream infections. *PLoS One* **6**, e21217, doi:10.1371/journal.pone.0021217 (2011).

188 Varona-Barquín, A., Iglesias-Losada, J. J., Ezpeleta, G., Eraso, E. & Quindós, G. Vancomycin heteroresistant community associated methicillin-resistant Staphylococcus aureus ST72-SCCmecIVa strain colonizing the nostrils of a five-year-old Spanish girl. *Enfermedades infecciosas y microbiologia clinica (English ed.)* **35**, 148-152 (2017).

189 Vellappally, S. *et al.* Occurrence of vancomycin-resistant Staphylococcus aureus in the oral cavity of patients with dental caries. *Acta microbiologica et immunologica Hungarica* **64**, 343-351 (2017).

190 Wang, J. L. *et al.* High vancomycin minimum inhibitory concentrations with heteroresistant vancomycin-intermediate Staphylococcus aureus in meticillin-resistant S. aureus bacteraemia patients. *Int J Antimicrob Agents* **42**, 390-394, doi:10.1016/j.ijantimicag.2013.07.010 (2013).

191 Whitener, C. J. *et al.* Vancomycin-resistant Staphylococcus aureus in the absence of vancomycin exposure. *Clinical Infectious Diseases* **38**, 1049-1055 (2004).

192 Wong, S. S.-Y. *et al.* Bacteremia due to Staphylococcus aureus with reduced susceptibility to vancomycin. *Diagnostic microbiology and infectious disease* **36**, 261-268 (2000).

193 Yamakawa, J. *et al.* Heterogeneously vancomycin-intermediate Staphylococcus aureus (hVISA) emerged before the clinical introduction of vancomycin in Japan: a retrospective study. *J Infect Chemother* **18**, 406-409, doi:10.1007/s10156-011-0330-2 (2012).

194 Yoon, J. Vancomycin resistance of Staphylococcus aureus in Korean primary hospitals. *Journal of Bacteriology and Virology* **44**, 305-310 (2014).

195 Yousefi, M. *et al.* Identification of tigecycline- and vancomycin-resistant Staphylococcus aureus strains among patients with urinary tract infection in Iran. *New Microbes New Infect* **19**, 8-12, doi:10.1016/j.nmni.2017.05.009 (2017).

196 Zeller, V., Kitzis, M.-D., Graff, W., Mamoudy, P. & Desplaces, N. Hip arthroplasty infection with heterogeneous vancomycin-resistant Staphylococcus aureus. *Scandinavian journal of infectious diseases* **38**, 934-938 (2006).

197 Zhang, X. *et al.* First report of a sequence type 239 vancomycin-intermediate Staphylococcus aureus isolate in Mainland China. *Diagnostic microbiology and infectious disease* **77**, 64-68 (2013).

198 Zhu, X. *et al.* Vancomycin intermediate-resistant Staphylococcus aureus (VISA) isolated from a patient who never received vancomycin treatment. *International Journal of Infectious Diseases* **33**, 185-190 (2015).

199 Lin, C.-Y., Wang, J.-H., Lin, K.-H., Ho, Y.-L. & Ho, C.-M. Methicillin-resistant Staphylococcus aureus with reduced vancomycin susceptibility in Taiwan. *Tzu-Chi Medical Journal* **30**, 135 (2018).

200 Institute, J. B. Joanna Briggs Institute reviewers’ manual: 2014 edition. *Australia: The Joanna Briggs Institute* (2014).

201 Mantel, N. & Haenszel, W. Statistical aspects of the analysis of data from retrospective studies of disease. *Journal of the national cancer institute* **22**, 719-748 (1959).

202 DerSimonian, R. & Laird, N. Meta-analysis in clinical trials. *Controlled clinical trials* **7**, 177-188 (1986).

203 Higgins, J. P., Thompson, S. G., Deeks, J. J. & Altman, D. G. Measuring inconsistency in meta-analyses. *Bmj* **327**, 557-560 (2003).

204 Gardete, S. & Tomasz, A. Mechanisms of vancomycin resistance in Staphylococcus aureus. *The Journal of clinical investigation* **124**, 2836-2840 (2014).

205 Cosgrove, S., Carroll, K. C. & Perl, T. Staphylococcus aureus with reduced susceptibility to vancomycin. *Clinical infectious diseases* **39**, 539-545 (2004).

206 Howden, B. P., Davies, J. K., Johnson, P. D., Stinear, T. P. & Grayson, M. L. Reduced vancomycin susceptibility in Staphylococcus aureus, including vancomycin-intermediate and heterogeneous vancomycin-intermediate strains: resistance mechanisms, laboratory detection, and clinical implications. *Clinical microbiology reviews* **23**, 99-139 (2010).

207 Walsh, T. R. & Howe, R. A. The prevalence and mechanisms of vancomycin resistance in Staphylococcus aureus. *Annual Reviews in Microbiology* **56**, 657-675 (2002).

208 Smith, T. L. *et al.* Emergence of vancomycin resistance in Staphylococcus aureus. *New England Journal of Medicine* **340**, 493-501 (1999).

209 Cui, L. *et al.* Cell wall thickening is a common feature of vancomycin resistance in Staphylococcus aureus. *Journal of clinical microbiology* **41**, 5-14 (2003).

210 Fridkin, S. K. *et al.* Epidemiological and microbiological characterization of infections caused by Staphylococcus aureus with reduced susceptibility to vancomycin, United States, 1997–2001. *Clinical Infectious Diseases* **36**, 429-439 (2003).

211 Bhattacharyya, D. *et al.* First report on vancomycin-resistant Staphylococcus aureus in bovine and caprine milk. *Microbial Drug Resistance* **22**, 675-681 (2016).

212 Moreno, L. Z. *et al.* Vancomycin-intermediate livestock-associated methicillin-resistant Staphylococcus aureus ST398/t9538 from swine in Brazil. *Memorias do Instituto Oswaldo Cruz* **111**, 659-661 (2016).

213 Ho, P.-L. *et al.* Vancomycin MIC creep in MRSA isolates from 1997 to 2008 in a healthcare region in Hong Kong. *Journal of Infection* **60**, 140-145 (2010).

214 Chang, W. *et al.* Vancomycin MIC creep in methicillin-resistant Staphylococcus aureus (MRSA) isolates from 2006 to 2010 in a hospital in China. *Indian journal of medical microbiology* **33**, 262 (2015).

215 Jacob, J. T. & DiazGranados, C. A. High vancomycin minimum inhibitory concentration and clinical outcomes in adults with methicillin-resistant Staphylococcus aureus infections: a meta-analysis. *International Journal of Infectious Diseases* **17**, e93-e100 (2013).

216 Van Hal, S., Lodise, T. P. & Paterson, D. L. The clinical significance of vancomycin minimum inhibitory concentration in Staphylococcus aureus infections: a systematic review and meta-analysis. *Clinical Infectious Diseases* **54**, 755-771 (2012).

217 Kim, T. *et al.* Clinical and microbiological factors associated with early patient mortality from methicillin-resistant Staphylococcus aureus bacteremia. *The Korean journal of internal medicine* **34**, 184 (2019).

218 Kelley, P. G., Gao, W., Ward, P. B. & Howden, B. P. Daptomycin non-susceptibility in vancomycin-intermediate Staphylococcus aureus (VISA) and heterogeneous-VISA (hVISA): implications for therapy after vancomycin treatment failure. *Journal of antimicrobial chemotherapy* **66**, 1057-1060 (2011).

219 Cui, L., Tominaga, E., Neoh, H.-m. & Hiramatsu, K. Correlation between reduced daptomycin susceptibility and vancomycin resistance in vancomycin-intermediate Staphylococcus aureus. *Antimicrobial agents and chemotherapy* **50**, 1079-1082 (2006).

220 Sieradzki, K., Roberts, R. B., Haber, S. W. & Tomasz, A. The development of vancomycin resistance in a patient with methicillin-resistant Staphylococcus aureus infection. *New England Journal of Medicine* **340**, 517-523 (1999).

221 Hageman, J. C. *et al.* Management of persistent bacteremia caused by methicillin-resistant Staphylococcus aureus: a survey of infectious diseases consultants. *Clinical Infectious Diseases* **43**, e42-e45 (2006).

222 McGuinness, W. A., Malachowa, N. & DeLeo, F. R. Focus: infectious diseases: vancomycin resistance in Staphylococcus aureus. *The Yale journal of biology and medicine* **90**, 269 (2017).

223 Siegel, J. Healthcare Infection Control Practices Advisory Committee 2007 Guideline for isolation precautions: preventing transmission of infectious agents in healthcare settings. [*http://www*](http://www)*. cdc. gov/ncidod/dhqp/gl_isolation. html* (2007).

224 Ayliffe, G. *et al.* Revised guidelines for the control of methicillin-resistant Staphylococcus aureus infection in hospitals: report of a combined working party of the British Society for Antimicrobial Chemotherapy, the Hospital Infection Society and the Infection Control Nurses Association. *Journal of Hospital Infection* **39**, 253-290 (1998).

225 Luzar, M. A. *et al.* Staphylococcus aureus nasal carriage and infection in patients on continuous ambulatory peritoneal dialysis. *New England Journal of Medicine* **322**, 505-509 (1990).

226 Di Gregorio, S. *et al.* Increase in IS256 transposition in invasive vancomycin heteroresistant Staphylococcus aureus isolate belonging to ST100 and its derived VISA mutants. *Infection, Genetics and Evolution* **43**, 197-202 (2016).
